# Prediction and impact of personalized donation intervals

**DOI:** 10.1111/vox.13223

**Published:** 2021-11-26

**Authors:** Jarkko Toivonen, Yrjö Koski, Esa Turkulainen, Femmeke Prinsze, Pietro della Briotta Parolo, Markus Heinonen, Mikko Arvas

**Affiliations:** ^1^ Finnish Red Cross Blood Service (FRCBS) Helsinki Finland; ^2^ Sanquin Research Amsterdam The Netherlands; ^3^ Institute for Molecular Medicine Finland Helsinki Finland; ^4^ Department of Computer Science Aalto University Helsinki Finland

**Keywords:** blood collection, donor health, haemoglobin measurement

## Abstract

**Background and Objectives:**

Deferral of blood donors due to low haemoglobin (Hb) is demotivating to donors, can be a sign for developing anaemia and incurs costs for blood establishments. The prediction of Hb deferral has been shown to be feasible in a number of studies based on demographic, Hb measurement and donation history data. The aim of this paper is to evaluate how state‐of‐the‐art computational prediction tools can facilitate nationwide personalized donation intervals.

**Materials and Methods:**

Using donation history data from the last 20 years in Finland, FinDonor blood donor cohort data and blood service Biobank genotyping data, we built linear and non‐linear predictors of Hb deferral. Based on financial data from the Finnish Red Cross Blood Service, we then estimated the economic impacts of deploying such predictors.

**Results:**

We discovered that while linear predictors generally predict Hb relatively well, they have difficulties in predicting low Hb values. Overall, we found that non‐linear or linear predictors with or without genetic data performed only slightly better than a simple cutoff based on previous Hb. However, if any of our deferral prediction methods are used to assign temporary prolongations of donation intervals for females, then our calculations indicate cost savings while maintaining the blood supply.

**Conclusion:**

We find that even though the prediction accuracy is not very high, the actual use of any of our predictors in blood collection is still likely to bring benefits to blood donors and blood establishments alike.

**Highlights**

- More refined prediction models, even using genetic data, have only slightly better accuracy than a simple baseline model.
- In our models, the effect of the donation interval on the haemoglobin level was too small to make donor‐specific donation intervals possible. However, assigning a temporary fixed‐term prolongation of the donation interval when deferral is predicted is likely to bring positive health effects in the vulnerable group of female donors under age 30.
- All prediction models we implemented lead to cost savings when used to determine a temporary fixed‐term prolongation of donation interval for females.

## INTRODUCTION

Deferring a person from donating due to low haemoglobin (Hb) can be demotivating for the donor, incurs extra costs to blood establishment and may indicate that a donor has donated blood too frequently causing negative health effects such as anaemia [1]. To mitigate these negative effects, it is beneficial to be able to predict the donor's Hb value at a given date or directly predict whether the Hb will be below the deferral limit.

Previously, Baart et al. used logistic regression with non‐linear predictors to predict low Hb deferrals [2, 3]. Subsequently, Nasserinejad [4] and Fokkinga [5] used Bayesian linear mixed models (LMMs) to predict Hb.

In this study, we aim to develop prediction methods for Hb/deferral to improve donor health and reduce costs due to deferrals without damaging the blood supply. We essentially reimplement the methods of Nasserinejad and Fokkinga and run them on our larger datasets with additional variables. To our knowledge, we are the first to use genetic information as explanatory variables and to estimate the blood supply and economic effects of deploying a low‐Hb deferral model. We also publish our model implementations to make it easier to build future research on our results.

## MATERIALS AND METHODS

The blood donation and blood product information of the Finnish Red Cross Blood Service (FRCBS) until 2020 was collected in the eProgesa database (MAKSYSTEM, Paris, France). Here, the eProgesa dataset contains the donation histories of Finnish blood donors from the last 20 years: 6,414,193 donation attempts from 940,831 donors. These data are collected at every blood donation event, and they contain information about the Hb value (pre‐donation point‐of‐care capillary finger‐prick sample) [6], time of day, donation location, type of donation and amount of blood collected.

**FIGURE 1:**
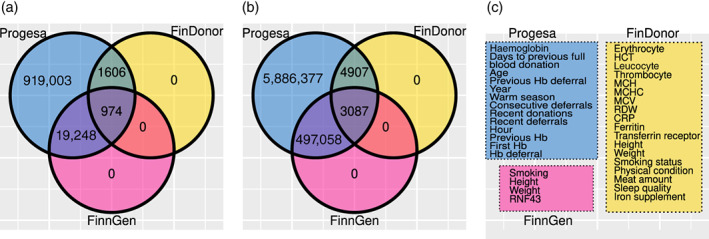
The intersections of the three datasets. (a) The number of donors and (b) the number of donations. The eProgesa dataset is shown in the raw form, before any pre‐processing was done. (c) The variables of each dataset

We pre‐processed the raw eProgesa data (Figure 1) to obtain a clean dataset for building models. Outliers, missing values and other problematic cases were handled by dropping instead of imputing them (Figure S2). We also derived several new variables from the raw variables (Figure 1c). After pre‐processing, we were left with 2,157,733 donations and 449,008 donors .

The Biobank dataset contains genome‐wide SNP genotyping data obtained from the Blood Service Biobank and height, weight and smoking variables from the Biobank enrolment questionnaire of 20,222 donors. The FinDonor [7] dataset contains more information about donation events such as blood counts, iron indices and questionnaire data. This dataset is much smaller than the eProgesa data, having a total of 7994 donation events from 2580 donors.

The variables from the eProgesa, Biobank and FinDonor datasets used for training our models are described in Tables S1–S3, respectively. Later in this paper, we refer to the combinations of eProgesa with the Biobank and FinDonor datasets with just Biobank and FinDonor, respectively. Further discussion about the variables used and pre‐processing can be found in Section S1.

As the donation history is a longitudinal dataset, we can apply LMMs (where some parameters can be stochastic instead of being fixed, as in normal linear models) to predict Hb. Our model has the form *y*_*it*_ = *x*_*it*_  *β* + *c*_*i*_  *ϕ* + *b*_*i*_ + *ε*_*it*_, where *i* refers to a donor and *t* to a donation time. The donation and donor‐specific variables are stored in matrices *x*_*it*_ and *c*_*i*_, respectively. The donor‐specific intercept *b*_*i*_ is the only random effect, and it allows deviation between donors caused by unobserved variables. If the previous Hb is among the predictors, then the model is called a dynamic linear mixed model (DLMM). Stan [8] is used to train these models in a Bayesian setting with weakly informative conjugate priors. To estimate the linear models' capability to predict deferral, the predicted Hb is dichotomized with the deferral limits used in Finland (135 g/L for men and 125 g/L for women).

To test whether the dependence of Hb is non‐linear with respect to the predictors, we use a random forest (RF) model [9]. Because deferrals are rare in Finnish donation history (approximately 3.2% of donations), in the RF algorithm, we oversample the deferrals so that the trees are created from samples where 50% of the donors have deferral as their last donation to make it easier to train a classifier for deferral. As an RF cannot directly model time series, we add to each donation event information about the previous Hb and the number of lifetime donations. We use randomForest [10] to train an RF whose hyperparameters were optimized with caret [11] using four‐fold cross‐validation. Details about the linear and RF models and their implementations can be found in Section S2.

We measured the accuracy of Hb prediction with root mean square error (RMSE) and mean absolute error (MAE) and the performance of the binary classifier of deferral with area under the receiver operating characteristic curve (AUROC), area under the precision‐recall curve (AUPR) and *F*‐score (F1) metrics. More details of the performance measures used can be found in Section S2.4.

Personalized donation intervals can be applied either by estimating a truly personal donation interval for each donor or by creating pre‐determined donation interval categories and assigning donors to them. In either case, the total adjustment *a*_tot_ in the population of returning donors is given by the mean of adjustments *a*_*i*_. If we extend the donation interval of donor *i* by 10%, for example, then *a*_*i*_ = 1.1. This adjustment has a direct inverse effect on the flow of returning donors, which we find by subtracting the influx of new donors from the total influx of donors. Thus, the total influx after adjustments is given by

$$F_{\mathrm{adj}}=\left(F-F_{\mathrm{new}}\right)/a_{\mathrm{tot}}+F_{\mathrm{new}}.$$

If donor recruitment efforts are not simultaneously increased, then the lowered influx is directly proportional to the supply level, for example, halving the total influx means halving the supply level, on average. Assuming that the supply level is held at the optimum before adjustments, the negative effects from donation interval personalization need to be compensated.

While marketing efficacy is not constant over long periods and while the cost of recruitment might increase with the size of the compensation, we can calculate estimates for the costs of this compensation for small enough adjustments by assuming a direct inverse relationship between the lowered influx of donors and the marketing efforts/budget:

$$M_{\mathrm{new}}=F/F_{\mathrm{adj}}\;\;M.$$

In addition to increased donor recruitment costs, we need to consider the savings from avoided deferrals after personalizing donation intervals, as deferring a donor may negatively impact donor retention. Avoiding deferrals decreases the negative impact on donor retention, which we otherwise would have had to compensate in marketing. The full marketing‐related economic effects of interval personalization can then be summarized as

$$E_{M}=F/F_{\mathrm{adj}}–1-\left(1-F_{\mathrm{new}}\right)\;d\;q\;r_{\text{loss}},$$

where we signify the negative impact of deferral on donor retention with *r*_loss_, the rate of avoided deferrals with *q* and the population deferral rate with *d*.

By finding the costs for marketing (per successful donation) and deferrals, we can expand this formulation into an equation that outputs the operational cost of deploying a type of personalization model in units of cost per donation:

$$E=P_{M}\;\left(F/F_{\mathrm{adj}}–1-\left(1-F_{\mathrm{new}}\right)\;d\;q\;r_{\text{loss}}\right)-P_{D}\;d\;q,$$

where *P*_M_ represents the estimated marketing cost of a single successful donation, and *P*_D_ is the cost of a deferred donor. The boundary for financial gain is then at *E* = 0, with *E* < 0 indicating savings and *E* > 0 costs incurred. The parameter values that apply to the FRCBS are listed in Table 1. Our economic effect formulation allows us to adjust for the model performance via the terms *a*_tot_ and *q*. A good model needs to extend the total donor influx only by very little to avoid most of the possible deferrals in the population (so *a*_tot_ = 1 + *ε*, 0 ≤ *ε* ≪ 1). If we let *a*_tot_ and *q* vary between chosen value ranges, then we can calculate the cost surface between these axes. Figure S38 presents the cost surface for the FRCBS drawn using values given in Table 1.

**TABLE 1:** The description of parameters in the cost effect formula and the parameter values specific to Finnish Red Cross Blood Service

Variable	Explanation	Value	Comment
*q*	Rate of avoided deferrals	[0,1]	From none to all.
*d*	Rate of deferrals in the population	0.032	From donor history data between 2018 and 2020.
*a*_tot_	Total adjustment effect due to interval extension	>1	
*P*_M_	Estimated marketing cost of a successful donation	2.287	Euros. An approximation based on the price of targeted and untargeted marketing per donation and response rates to targeted marketing.
*P*_D_	Cost of a deferred donor	20.342	Euros. Comprises costs of materials, marketing and work time.
*F*	Total influx of donors	1	As in 100%.
*F*_new_	Influx of new donors	0.107	Currently, new donors comprise about 10% of the donor influx.
*r*_loss_	Impact of deferral on donor retention	0.167	Low Hb deferrals are currently estimated to have approximately 16.7% negative impact on donor retention. This analysis is detailed in Section S3.3.1.

## RESULTS

To determine, which subset of full data are best suited as the input for fitting LMMs, we performed three experiments: effect of time series length, effect of amount of data and effect of the imbalance of the division of the donations into accepted and deferred classes on Hb prediction. In addition, to determine whether the rules for selecting the input subset generalize, we divided the data into two equal‐sized halves: an exploration part and a final model fitting and testing part. The three experiments described below were all performed on the exploration part of the data.

The distribution of time‐series length of female donors in eProgesa data is shown in Figure S8. The number of donors decreases exponentially as a function of the time‐series length. To model the data and predict the last donation of each time series, the minimum theoretical time series length is three. This requirement already dropped 50% of the donors from further consideration. To test the effect of the time‐series length on Hb prediction, we partitioned the female eProgesa data into subsets based on time‐series length, with each subset having donors with the same number of donations. We fitted a DLMM on each of these datasets and predicted the Hb of the last donation of the time series. The results are shown in Table S6. On the one hand, the results seemed to improve as the time‐series length increased. On the other hand, the data were scarcer with longer time series. As a compromise, we decided to use the data from donors with at least seven donations in our later analyses of eProgesa and Biobank data.

We also experimented with the effect of the amount of data on the prediction. We randomly took three samples from female eProgesa data with 10,000, 30,000 and 50,000 donors. The amount of data did not show any clear effect on the prediction results (see Table S7).

Next, we considered the imbalance between accepted and deferred donation classes. In the pre‐processed eProgesa data, only 12% of the donors had at least one deferral and the number of donors with more deferrals decreased rapidly, as shown in Figure S9. Since we want to be able to accurately predict Hb values that are below the accepted threshold, it is vital that there are enough examples of low Hb donations in the training data. We tried to artificially enrich the fraction of deferrals by dropping out donors with no deferrals. This resulted in a subset of female eProgesa data with the fraction of donors with at least one deferral being 50%. We fitted a DLMM on these data, and the prediction results are shown in Table S8. The results worsened after the enrichment.

As a result of the above exploration, we decided not to enrich the training data used for fitting LMMs. In addition, we only included donors who had donated at least seven times. The resulting dataset was already small enough, so fitting a model on these data was feasible in terms of time and memory required. Hence, no further subsetting of the data was needed. The resulting final testing data had 695,658 donations (398,803 female and 296,855 male) from 47,820 donors (29,298 female and 18,522 male). This dataset was used in the final analyses of the eProgesa data and the Biobank data. A different scheme, explained in Section S3.2.4, was used for FinDonor data, as that data had a very small number of donors and donations.

**FIGURE 2:**
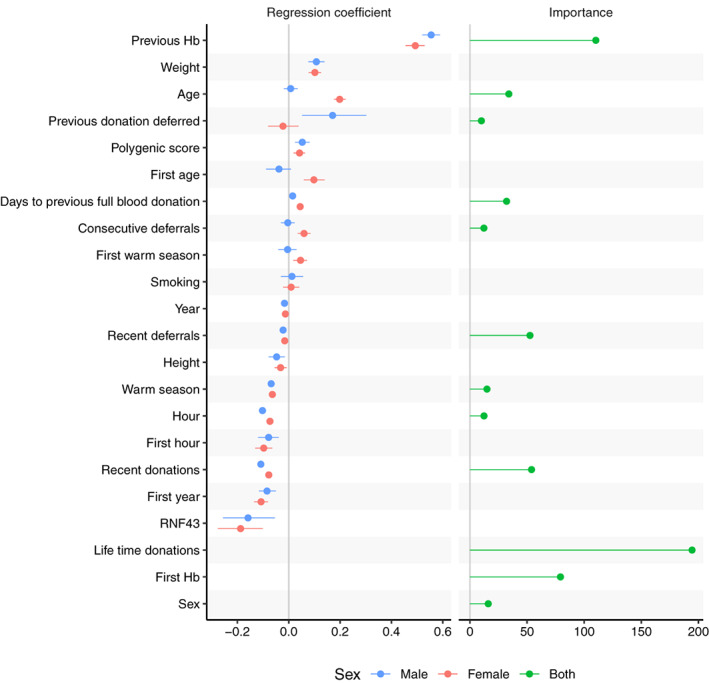
The effect sizes and importance of variables in haemoglobin and deferral prediction. On the left panel, the regression coefficients of the DLMM when predicting haemoglobin on variables of the combined eProgesa and Biobank data. The dots and the lines denote the posterior means and the 95% highest posterior density intervals (HPDIs), respectively, for each variable and sex. In order to make the regression coefficients comparable, we left the binary variables as they are but scaled other variables by 2  SD. Hence, the units of the regression coefficients are two times the standard deviation. On the right, the importance of variables, when predicting deferral using a random forest model on eProgesa data, are marked with dots. With the random forest model, we did not train separate models for the male and female subsets but instead used sex as a predictor. Note that for both DLMM and random forest (RF) models the previous haemoglobin was clearly the most important variable. The difference of effect size between sexes seems to be mostly small, the age being a notable exception

Figure 2 illustrates the effect sizes of variables of Biobank data in DLMM and the importance of eProgesa variables in RF. Other models gave similar results (Figures S10, S11, S20, S26, S31 and S34). There were no large differences in effect sizes between men and women except for the age‐related variables. In both models, previous Hb was clearly the most important variable. The SNP rs199598395 on gene RNF43 had a large influence, but a polygenic score of Hb calculated from UK Biobank data had a smaller effect size than whether the donation was given in April–September.

The effect of the “days to previous full blood donation” was so small that varying it and other time‐dependent variables accordingly did not affect deferral prediction enough to enable fully personalized donation intervals. Hence, we analysed the effect of donation activity by demographic group on the low Hb deferral rate for all donations in Finland (Figure S6) and the effect of donation on the iron deficiency rate in FinDonor data (Figure S7). Although no clear association existed between deferral rate and donation activity, a fixed deferral of 12 months is likely to reduce deferrals in the most vulnerable group, that is, women younger than 30 (Figure S6). For ferritin levels of the same group, even a 6‐month deferral would decrease the number of yearly donations by up to two or three and hence significantly decrease the prevalence of low iron (Figure S7). In general, a 6‐month interval, possibly with supplemental iron, has previously been shown to allow ferritin recuperation for most donors [12, 13].

To estimate the economic effects of deferral prediction, we subsequently used these two alternative donation intervals in case a model predicted a donor to be deferred. When we temporarily extended the donation intervals of all true and predicted deferrals to either 6 or 12 months, we obtained a rough but concrete estimate of the cost effect of the model performance.

**FIGURE 3:**
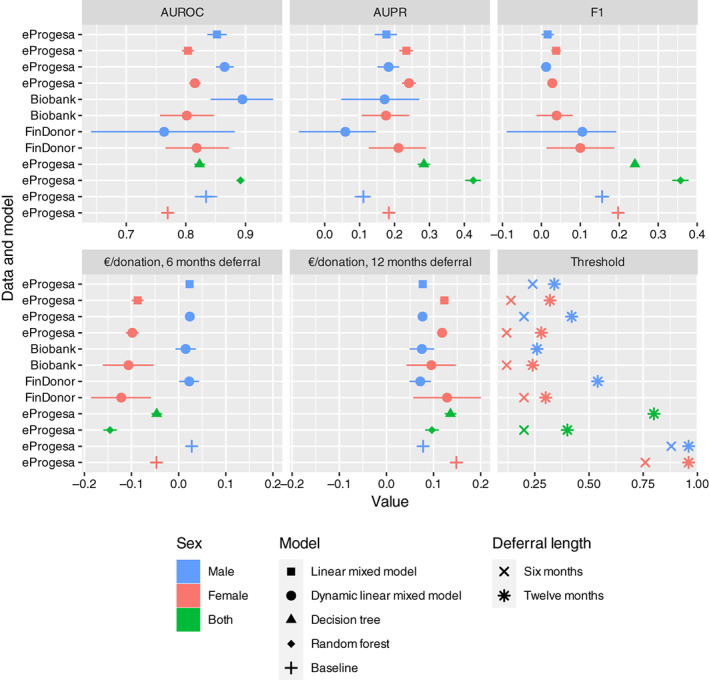
Performance metrics and economic effects for the models. The AUROC, AUPR and F1 are standard metrics measuring the performance of a binary classifier. We also show the economic effect of assigning either a 6‐ or 12‐month donation interval to donors who are predicted to be deferred. A negative effect means savings in units of euro per donation. From the Hb prediction of the Bayesian linear mixed models, we calculate the probability of deferral based on Finnish deferral limits, while the random forest model outputs probabilities of deferrals directly. To calculate economic effects, these probabilities of deferrals need to be dichotomized into a deferral status by a cutoff value. The threshold panel shows, which cutoff for the probability of deferral, applied to a given model, gave the optimal savings (shown on the economic effect panels), where the candidates for cutoffs were 0.02, 0.04, …, 0.98. For all the panels except the threshold panel, 95% confidence intervals computed using bootstrapping are shown. Most of the savings come from avoiding female deferrals

The deferral prediction results and economic effects are summarized in Figure 3 and Table S14. According to the AUROC metric, RF performed better than the other models except for the male DLMM with Biobank data. However, there were no large differences in the performance of the LMM and RF, and each was only slightly better than our baseline model, that is, logistic regression with previous Hb as the only predictor.

In addition, all female models and the RF model resulted in cost reductions when a 6‐month deferral was applied for those predicted to be deferred (Figure 3). For RF, the economic effect was −0.15 euro per donation, that is, an economic savings of 0.15 euro per donation, and the average interval extension was 1.1. Deployment of this model would result in avoiding 51% of the deferrals. In the models that were trained by stratifying by sex, the average cost effect for males was 0.02 euro per donation, whereas, for women, the effect was −0.11 euro per donation. If both male and female models were applied, then the DLMM with FinDonor data gave the second‐largest savings at approximately 0.1 euro per donation, with the average donation interval length being 1.12‐fold. This model enabled us to avoid 51% of deferrals.

As the baseline model predicts based on previous Hb only, the probability thresholds that were found to provide the largest savings correspond to specific Hb values. For 6‐month deferral, these were 147 g/L for men and 135 g/L for women; and for 12‐month deferral, 141 g/L for men and 122 g/L for women.

## DISCUSSION

**FIGURE 4:**
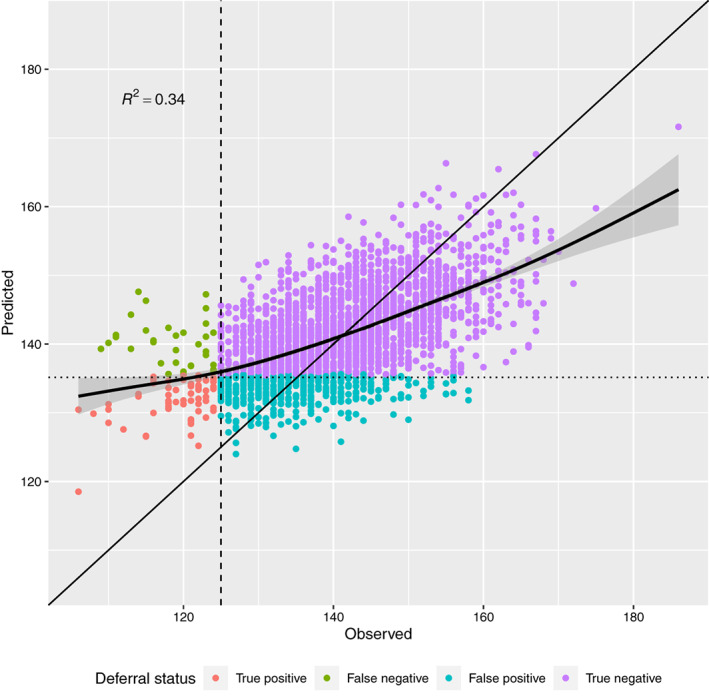
The observed and predicted Hb values given by the Biobank female DLMM are plotted with the *R* ^2^ correlation value. The fitted smooth curve (generalized additive model) and its 95% confidence intervals show the difficulty in predicting extreme Hb values. The donations are classified into deferred (positive) and accepted (negative) classes first by comparing the observed values and the standard female threshold 125 g/L (dashed vertical line). Second, the probability of the haemoglobin deferral is compared to the 6‐month deferral probability cutoff of 0.12 (found by maximizing savings, see Figure 3 panel threshold), which corresponds roughly to the predicted Hb value 135.2 g/L (dotted horizontal line). Note that had we used the same threshold for predicted Hb as for observed Hb (125 g/L), we would have been able to predict only one deferral correctly

Our reimplementation of the LMMs gives equal or slightly weaker results in terms of MAE and RMSE (Table S13) but better results in terms of AUROC than in Fokkinga [5]. This is probably due to larger data and more variables, but these approaches still fail to predict lower Hb values. For example, the female DLMM on Biobank data predicts for all but one donation where Hb is below 125 g/L higher than 125 g/L Hb. However, if the deferral threshold that gives optimal economic effects is used instead, then we can avoid 49% of the deferrals while falsely predicting as deferred only 18% of the viable donations (Figure 4). We expect the prediction results to be more accurate in countries where the deferral rate is higher than in Finland since the ratio of accepted and deferred donations is more balanced. Although the incorporation of genetic information as predictors improves the prediction (Figure 3, Table S14), the effect appears small in relation to the costs of genotyping. The SNP rs199598395 in the RNF43 gene was discovered by the FinnGen project as a lead SNP for iron deficiency anaemia (http://r4.finngen.fi/pheno/D3_ANAEMIA_IRONDEF). Its effect size is large, but the minor allele is only present in ~2% of donors. Overall, in Finns, it is present in ~1% of people, in Europeans (non‐Finnish) ~0.01% and it is not found in other populations [14]. This highlights the possibility that further study of population‐specific or rare genetic variation could considerably increase the value of genetic predictors.

Our RF model performs similarly to logistic regression with non‐linear predictors [3] in predicting deferral but is simpler and easier to train. There is no apparent performance difference between the LMMs and RF in predicting deferral. Importantly, these complicated models seem to have little benefit over a simple one‐predictor logistic regression (baseline model).

Due to the low accuracy in Hb prediction and the fact that the effect of the “days to previous full blood donation” variable is small, we were unable to define completely personalized donation intervals. However, our calculations on the blood supply and economic effects indicate that cost reduction is still possible through a fixed deferral (6 months) given to donors (especially female donors) predicted to be deferred. To our knowledge, this is the first report that estimates the blood supply and economic effects of deploying a deferral prediction model. However, our calculations are based on two assumptions: (1) that every euro spent on marketing will result in a proportional number of new donors coming in and (2) that the Hb values recover as a function of time. Although assumption (1) is certainly not universally valid, we believe that it is very likely to be valid for the small adjustments we make here.

In conclusion, our results suggest that pre‐donation Hb data could be used much more efficiently to bring savings and health benefits. Furthermore, savings to donors will result in saved time and travel expenses [15], although we did not include them in our estimation. If the pre‐donation Hb value is found to be below the threshold for economic effects but above the deferral limit, then the donor can donate but is deferred, for example, for 6 months. We do not find that the more complicated computational predictors could greatly improve on this. However, more predictive data such as ferritin measurements at every donation, more informative genetic data, or iron consumption and menstruation data could bring significant improvements. We have started evaluating the deployment of the threshold‐based system at the FRCBS. This includes assessing the effect of varying the cost parameters, risk analysis and possible testing of the procedure at a single donation site.

**FIGURE 5:**
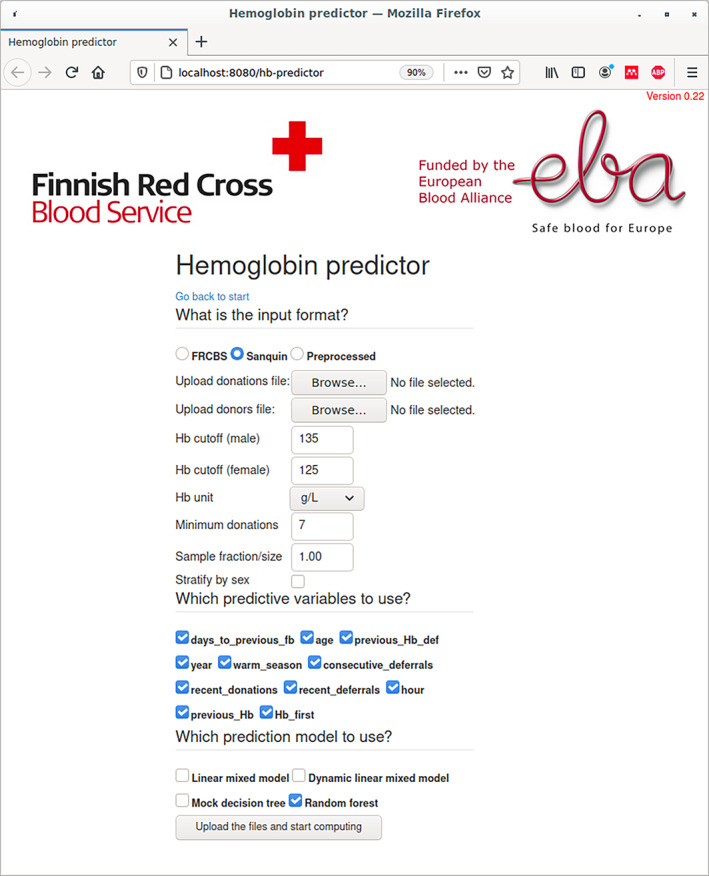
The user interface to the prediction models works in any web browser. After the parameters are configured and the input data are uploaded, the computation begins. The result is given as both html and pdf documents that contain plots and tables, with the possibility of downloading the results in a raw form for further processing

The source code of the model implementations is available at GitHub (see Supporting Information for details) and Zenodo [16]. Furthermore, a ready‐to‐use prediction application as a Docker [17] software container is also provided. Its user interface, which runs in a web browser, facilitates easy use for non‐programmers (see Figure 5). These resources allow others to test our models with their data and develop them further; see Section S2.3 for more details.

## CONFLICT OF INTEREST

There are no conflicts identified.

## Supporting information


**Appendix S1** Supporting Information

## References

[1] Custer B , Chinn A , Hirschler NV , Busch MP , Murphy EL . The consequences of temporary deferral on future whole blood donation. Transfusion. 2007;47:1514–23. 17655597 10.1111/j.1537-2995.2007.01292.x

[2] Baart AM , De Kort WLAM , Moons KGM , Vergouwe Y . Prediction of low haemoglobin levels in whole blood donors. Vox Sang. 2011;100:204–11. 20726956 10.1111/j.1423-0410.2010.01382.x

[3] Baart AM , De Kort WLAM , Atsma F , Moons KGM , Vergouwe Y . Development and validation of a prediction model for low hemoglobin deferral in a large cohort of whole blood donors. Transfusion. 2012;52:2559–69. 22519683 10.1111/j.1537-2995.2012.03655.x

[4] Nasserinejad K . Modeling longitudinal data of blood donors. PhD dissertation. Erasmus University Rotterdam. 2016.

[5] Fokkinga J. Modelling hemoglobin levels of blood donors. Master's thesis. Erasmus University Rotterdam. 2018.

[6] Bäckman S , Larjo A , Soikkeli J , Castrén J , Ihalainen J , Syrjälä M . Season and time of day affect capillary blood hemoglobin level and low hemoglobin deferral in blood donors: analysis in a national blood bank. Transfusion. 2016;56:1287–94. 27018648 10.1111/trf.13578

[7] Lobier M , Niittymäki P , Nikiforow N , Palokangas E , Larjo A , Mattila P , et al. FinDonor 10 000 study: a cohort to identify iron depletion and factors affecting it in Finnish blood donors. Vox Sang. 2020;1:36–46. 10.1111/vox.12856 PMC7004091 31657023

[8] Carpenter B , Gelman A , Hoffman MD , Lee D , Goodrich B , Betancourt M , et al. Stan: a probabilistic programming language. J Stat Softw. 2017;76:1–32. 10.18637/jss.v076.i01 PMC9788645 36568334

[9] Breiman L . Random forests. Mach Learn. 2001;45:5–32.

[10] Liaw A , Wiener M . Classification and regression by randomForest. R News. 2002;2:18–22.

[11] Kuhn M caret: classification and Regression Training [Internet]. 2020. [Cited 2021 Oct 5]. Available from: https://cran.r-project.org/package=caret

[12] Kiss JE , Brambilla D , Glynn SA , Mast AE , Spencer BR , Stone M , et al. Oral iron supplementation after blood donation: a randomized clinical trial. JAMA. 2015;313:575–83. 25668261 10.1001/jama.2015.119 PMC5094173

[13] Schotten N , Pasker‐de Jong PCM , Moretti D , Zimmermann MB , Geurts‐Moespot AJ , Swinkels DW , et al. The donation interval of 56 days requires extension to 180 days for whole blood donors to recover from changes in iron metabolism. Blood. 2016;128:2185–8. 27587880 10.1182/blood-2016-04-709451

[14] Karczewski KJ , Francioli LC , Tiao G , Cummings BB , Alföldi J , Wang Q , et al. The mutational constraint spectrum quantified from variation in 141,456 humans. Nature. 2020;581:434–43. 32461654 10.1038/s41586-020-2308-7 PMC7334197

[15] de Kort W , van den Burg P , Geerligs H , Pasker‐de Jong P , Marijt‐van der Kreek T . Cost‐effectiveness of questionnaires in preventing transfusion‐transmitted infections. Transfusion. 2014;54:879–88. 23889559 10.1111/trf.12349

[16] Toivonen J , Koski Y , Arvas M . Software for the article Prediction and impact of personalised donation intervals. 2021. 10.5281/zenodo.5549879#.YVw31tABAhp.mendeley PMC9299493 34825380

[17] Docker Inc . Docker 2020 [Internet]. [cited 2021 Oct 5]. Available from: https://www.docker.com/

