# Supplementary material for: Prediction and impact of personalized donation intervals
Source: Vox Sang. 2021 Nov 26;117(4):504–12. doi: 10.1111/vox.13223 (PMC9299493; doi:10.1111/vox.13223)
Supplement: Supplementary file 1 — Appendix S1 Supporting Information [file VOX-117-504-s001.pdf]

Prediction and impact of personalized donation intervals
Supplementary material

November 1, 2021

Finnish Red Cross
Blood Service

Funded by the
European
Blood Alliance
eba
Safe blood for Europe

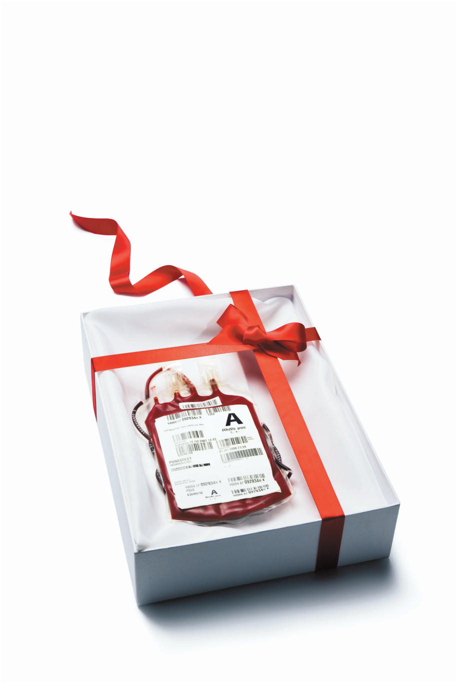

Contents

S1 Data details **S5**
S1.1 eProgesa data set . . . S5
S1.2 Biobank data set . . . S7
S1.3 FinDonor data set . . . S9

S2 Methods details **S12**
S2.1 Linear Mixed Model (LMM) . . . S12
S2.1.1 Dynamic linear mixed model (DLMM) and the initial conditions problem . . . S12
S2.1.2 Incorporating individual-specific variables . . . S13
S2.1.3 Bayesian framework . . . S13
S2.1.4 Prior specifications . . . S13
S2.1.5 Out-of-sample predictions . . . S14
S2.1.6 Dynamic predictions . . . S14
S2.2 Random forest . . . S15
S2.3 Model implementations . . . S15
S2.4 Performance metrics . . . S16

S3 Results details **S18**
S3.1 Definition of alternative donation intervals . . . S18
S3.2 Development of computational predictors . . . S21
S3.2.1 Exploring data . . . S21
S3.2.2 Linear mixed models on eProgesa data . . . S22
S3.2.3 Linear mixed models on Biobank data . . . S30
S3.2.4 Linear mixed models on FinDonor data . . . S34
S3.2.5 Random forest on eProgesa data . . . S39
S3.3 Estimation of financial and blood supply effects . . . S44
S3.3.1 Estimation of the effect of Hb deferral on donor retention . . . S44
S3.3.2 Cost effect at the FRCBS . . . S44

References **S48**

List of Tables

S1 Description of variables in the eProgesa data set . . . S5
S2 Description of the additional donor specific variables in the Biobank data set . . . S7
S3 Description of additional variables in the FinDonor data set . . . S9
S4 Linear mixed models written in Stan for in-sample-prediction and their short descriptions. . . . S16
S5 Linear mixed models written in Stan for out-of-sample prediction and their short descriptions. . S16
S6 The effect of time series length . . . S21
S7 The effect of the number of donors on prediction . . . S22
S8 The effect of enrichment of deferrals in female eProgesa data . . . S23
S9 The effect sizes in a LMM on eProgesa data . . . S24
S10 The effect sizes in a DLMM on eProgesa data . . . S25
S11 The effect sizes in a DLMM on Biobank data . . . S31
S12 The effect sizes in a DLMM on FinDonor data . . . S36
S13 Hemoglobin prediction errors for each data set and model . . . S46
S14 The numeric values of the classification performance metrics . . . S47

List of Figures

S1 Summary plots of the preprocessed eProgesa variables . . . S6
S2 Exclusions during preprocessing of the eProgesa data . . . S6
S3 Summary plots of the Biobank variables . . . S8
S4 Summary plots of the donation specific FinDonor variables . . . S10
S5 Summary plots of the donor specific FinDonor variables . . . S11
S6 Effect of previous donation activity on deferral rate . . . S18
S7 Bootstrapped effect of donation activity on iron deficiency rates of women . . . S19
S8 The distribution of time series length of female donors in eProgesa data set . . . S21
S9 Distribution of time series length in female eProgesa data . . . S22
S10 Effect sizes in a LMM on eProgesa data . . . S23
S11 Effect sizes in a DLMM on eProgesa data . . . S24
S12 Scatter plot and confusion matrix in female eProgesa data by a LMM . . . S26
S13 Scatter plot and confusion matrix in female eProgesa data by a DLMM . . . S26
S14 Scatter plot and confusion matrix in male eProgesa data by a LMM . . . S27
S15 Scatter plot and confusion matrix in male eProgesa data by a DLMM . . . S27
S16 Classification performance curves in female eProgesa data by a LMM . . . S28
S17 Classification performance curves in female eProgesa data by a DLMM . . . S28
S18 Classification performance curves in male eProgesa data by a LMM . . . S29
S19 Classification performance curves in male eProgesa data by a DLMM . . . S29
S20 Effect sizes in Biobank data by a DLMM . . . S30
S21 Scatter plot and confusion matrix in female Biobank data by a DLMM . . . S31
S22 Scatter plot and confusion matrix in male Biobank data by a DLMM . . . S32
S23 Deferral prediction using mean of posterior hemoglobin in male Biobank data with DLMM . . . S32
S24 Classification performance curves in female Biobank data by a DLMM . . . S33
S25 Classification performance curves in male Biobank data by a DLMM . . . S33
S26 Effect sizes in FinDonor data by a DLMM . . . S35
S27 Scatter plot and confusion matrix in female FinDonor data by a DLMM . . . S37
S28 Scatter plot and confusion matrix in male FinDonor data by a DLMM . . . S37
S29 Classification performance curves in female FinDonor data by a DLMM . . . S38
S30 Classification performance curves in male FinDonor data by a DLMM . . . S38
S31 Confusion matrix and variable importances in eProgesa data by the decision tree model . . . S39
S32 Classification performance curves in eProgesa data by the decision tree . . . S40
S33 Decision tree on eProgesa data . . . S40
S34 Confusion matrix and variable importances of the random forest model on eProgesa data . . . S41
S35 Classification performance curves in eProgesa data by the random forest . . . S42
S36 Density distributions of all predictor variables for females in random forest . . . S43
S37 Effect of an Hb deferral on donation frequency . . . S44
S38 Economical cost surface . . . S45
S39 Cost effect of the random forest . . . S46

S1 Data details

In this study we have used three partly overlapping data sets. Each of these are described in more detail in the upcoming subsections. The models are fitted using one data set or a combination of multiple sets. Details of the models are described in methods section.

S1.1 eProgesa data set

The eProgesa data set contains the donation histories of Finnish blood donors from the last 20 years. This data is collected at every blood donation event and it contains information about the hemoglobin value, time of day, location where the donation was collected, type of donation and the amount of blood collected.

We preprocessed the raw eProgesa data in order to get a clean data set for building models. Outliers, missing values, and other problematic cases were mostly handled by dropping them, no imputation of missing values was done. This results in large amount of data being left out from further analysis (Figure S2). Notably, over three million events are dropped because the date of first donation of the donor is not known. In the preprocessing we also derived several new variables from the raw variables. The resulting variables, that we used in the analyses, are described in Table S1 and summarized in Figure S1. After the preprocessing we were left with 2 157 733 donations and 449 008 donors.

Table S1: Description of variables in the eProgesa data set.

**Variable**	**Type**	**Explanation**
Donor ID	Factor	Donor identifier
Hemoglobin	numeric	Amount of Hemoglobin
Days to previous full blood donation	numeric (int)	Time (in days) between Hb measurement and previous full blood donation event
Age	numeric	Age of donor
Previous Hb deferral	boolean	Indicates whether the donor was deferred from blood donation due to low hemoglobin at previous donation event
Year	numeric (int)	Year of donation
Warm season	boolean	True if donation was given in April-September
Consecutive deferrals	numeric (int)	Amount of times the donor has been deferred due to low hemoglobin since last successful whole blood donation
Recent donations	numeric (int)	Amount of donations in the last two years
Recent deferrals	numeric (int)	Amount of deferrals due to low hemoglobin in the last two years
Hour	numeric	Time of day when donation was given as hours (e.g. 13:45 = 13.75)
Previous Hb	numeric	Hb value at previous measurement (ICP-model)
First Hb	numeric	Hb value at first donation of this donor (Non ICP-model)
Hb deferral	boolean	Deferred based on low hemoglogin

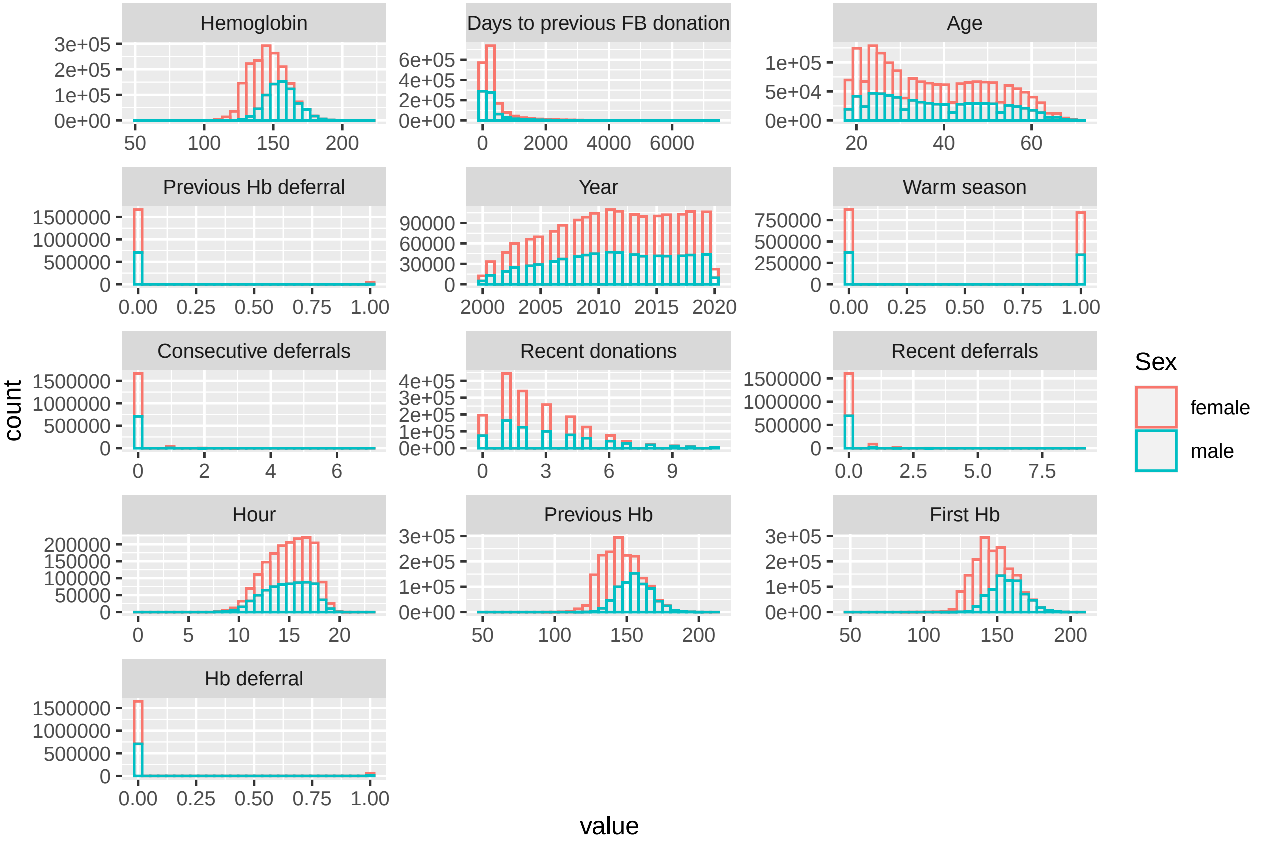

Figure S1: Summary plots of the thirteen variables in the preprocessed eProgesa data used in the linear models.

```
Dropped  612408 / 6414193 donations (175143 / 940831 donors) due to badly formed ID
Dropped    5734 / 5801785 donations (  1789 / 765688 donors) due to indentical date and dob
Dropped       0 / 5796051 donations (     0 / 763899 donors) due to date or dob being NA
Dropped    6228 / 5796051 donations (  3945 / 763899 donors) due to date '19390101'
Dropped 3286139 / 5789823 donations (246248 / 759954 donors) because date_first_donation
                                                             is not known.
Dropped      54 / 2503684 donations (    39 / 513706 donors) due to age at time of donation
Dropped   15473 / 2503630 donations (     0 / 513667 donors) because only last try of the day
                                                             is selected
Dropped  102030 / 2488157 donations ( 15305 / 513667 donors) because the given
                                                             date_first_donation was not the oldest
                                                             donation for that donor
Dropped  119567 / 2386127 donations ( 46630 / 498362 donors) whose first Hb is NA
Dropped   26358 / 2266560 donations (  2235 / 451732 donors) because donat_phleb was not 'K'
                                                             nor '*'
Dropped   82469 / 2240202 donations (   489 / 449497 donors) because Hb or days_to_previous_fb
                                                             was NA for a non-first donation
Final preprocessed data has 2157733 donations and 449008 donors
```

Figure S2: The number of donations and donors that were dropped in each phase during the preprocessing of the eProgesa data.

Table S2: Description of the additional donor specific variables in the Biobank data set.

Variable	Type	Description
Smoking	boolean	Does the person smoke currently
Height	numeric	Height of the donor
Weight	numeric	Weight of the donor
RNF43 minor allele	boolean	Presence of minor allelle at RNF43 gene in chromosome 17 position 58358769
Polygenic score	numeric	Polygenic risk score for hemoglobin

S1.2 Biobank data set

The Biobank data set contains genetic information about the donors and in addition three variables from the biobank enrollment questionnaire. This data set contains information for approximately 20 000 donors. These variables are described in Table S2.

To calculate hemoglobin polygenic scores for each individual in the data set, hemoglobin GWAS (genome wide association analysis) summary statistics were retrieved from `http://www.nealelab.is/uk-biobank/`. PRS weights were then calculated with PRS-CS [Ge+19]. The EUR reference panel provided with PRS-CS was used in the analysis, as its ancestry is closest to the used GWAS summary statistic data. Default parameters were used in PRS-CS i.e. the algorithm optimizes its parameters automatically. The final set of variants used for the scores is the intersection between the variants of the reference panel, the hemoglobin GWAS original summary statistics and the Biobank data. Finally, scores were calculated using plink2 [Cha+15], with the `--score` flag using the `center` option, which translates all dosages to mean zero.

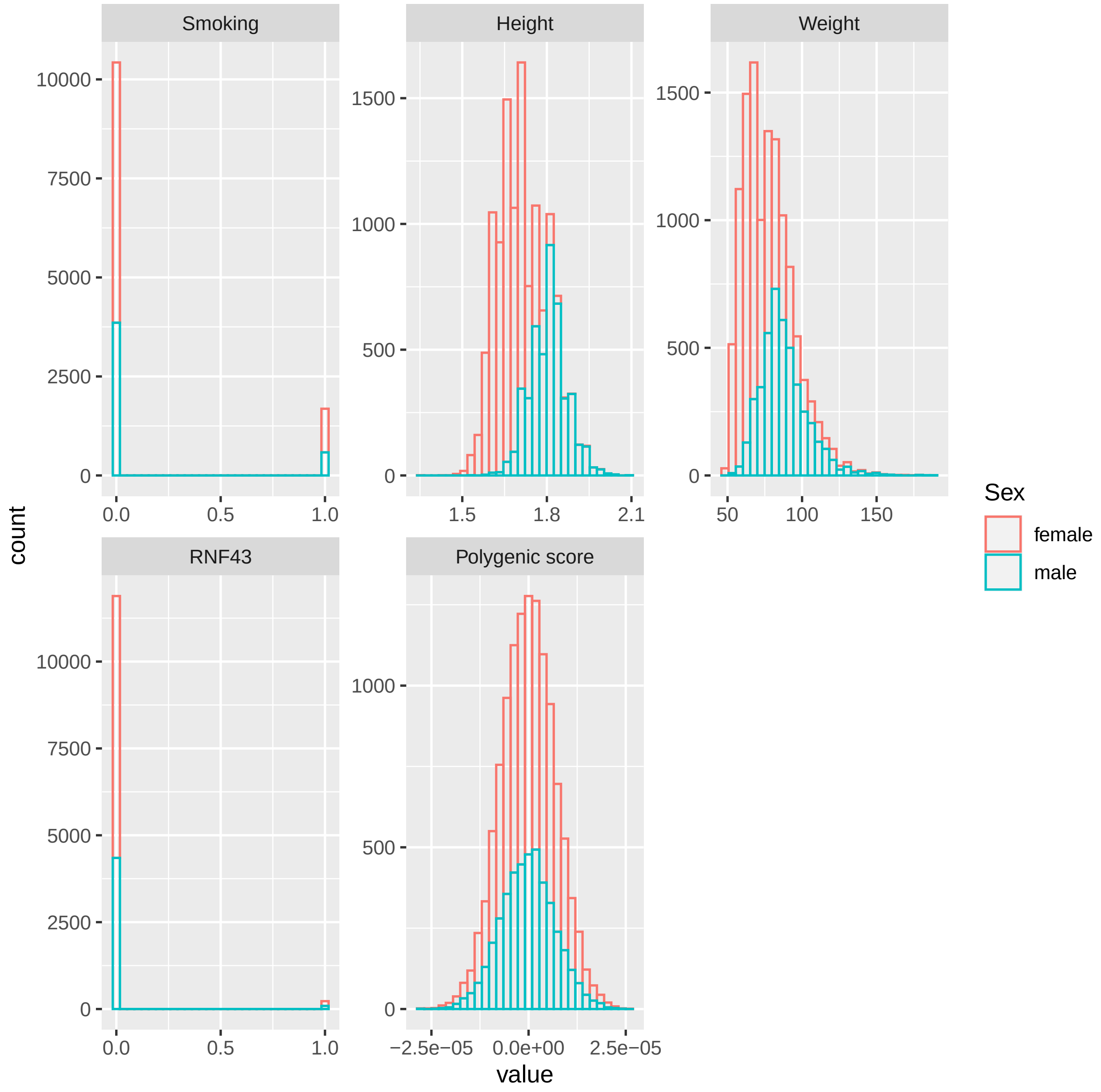

Figure S3: Summary plots of the five variables in the Biobank data used in the linear models.

S1.3 FinDonor data set

FinDonor data set [Lob+20] contains more information about donation events such as blood counts, iron indices and questionnaire data. The data set is much smaller than the eProgesa data having a total of 7994 donation events from 2580 donors. The additional variables from the FinDonor data, we used in our models, are described in Table S3. Of the 92 questionnaire items in the FinDonor data, we chose only relevant additional variables based on previous research [Lob+19; Fok18; Nas16; Bäc+20; Cus+14; Kot+15]. Distributions of the donation specific variables are shown in Figure S4 and for donor specific variables S5.

Table S3: Description of additional variables in the FinDonor data set.

Variable	Type	Description
Erythrocyte	Numeric	Amount of erythrocytes, red blood cells [E12/l]
HCT	Numeric	Hematocrit (HCT) the volume percentage of RBCs in whole blood [%]
Leukocyte	Numeric	Leukocytes, white blood cell counts [E9/l] (thousand cells per microliter)
Thrombocyte	Numeric	Thrombocytes/platelets [E9/l]
MCH	Numeric	Mean corpuscular hemoglobin, average mass of hemoglobin per red blood cell. Diminished in hypochromic anemias. [pg]
MCHC	Numeric	Mean corpuscular hemoglobin concentration, average mass of hemoglobin in a litre of RBCs. [g/l]
MCV	Numeric	Mean Corpuscular Volume, the average volume of RBCs. [fl]
RDW	Numeric	Red blood cell distribution width, is a measure of the range of variation of RBC volume. [%]
CRP	Numeric	C-reactive protein, measure of inflammation. Its circulating concentrations rise in response to inflammation. [mg/l]
Ferritin	Numeric	An intracellular protein that stores iron and releases it in controlled fashion. Acts as a buffer against iron deficiency and iron overload. Plasma ferritin is an indirect marker of the total iron storage. [µg/l]
Transferrin receptor	Numeric	Transferrin receptor (TfR) is a carrier protein for transferrin. [mg/l]
Height	Numeric	Height
Weight	Numeric	Weight
Smoking status*	Boolean	Smoking status: no=0, sometimes or daily = 1
Physical condition*	Numeric (int)	Physical condition: very bad=0, bad=1, satisfactory=2, rather good=3, good=4, excellent=5
Meat amount*	Numeric (int)	How often meat: less than one weekly=1, 1-3 per week=2, 4-6 per week=3, daily=4, several daily=5
Sleep quality*	Numeric (int)	How often do you feel like you've slept enough: never=0, rarely = 1, mostly=2, always or almost = 3
Iron supplement*	Numeric (int)	How many iron table were eaten

* These values are gathered from a questionnaire

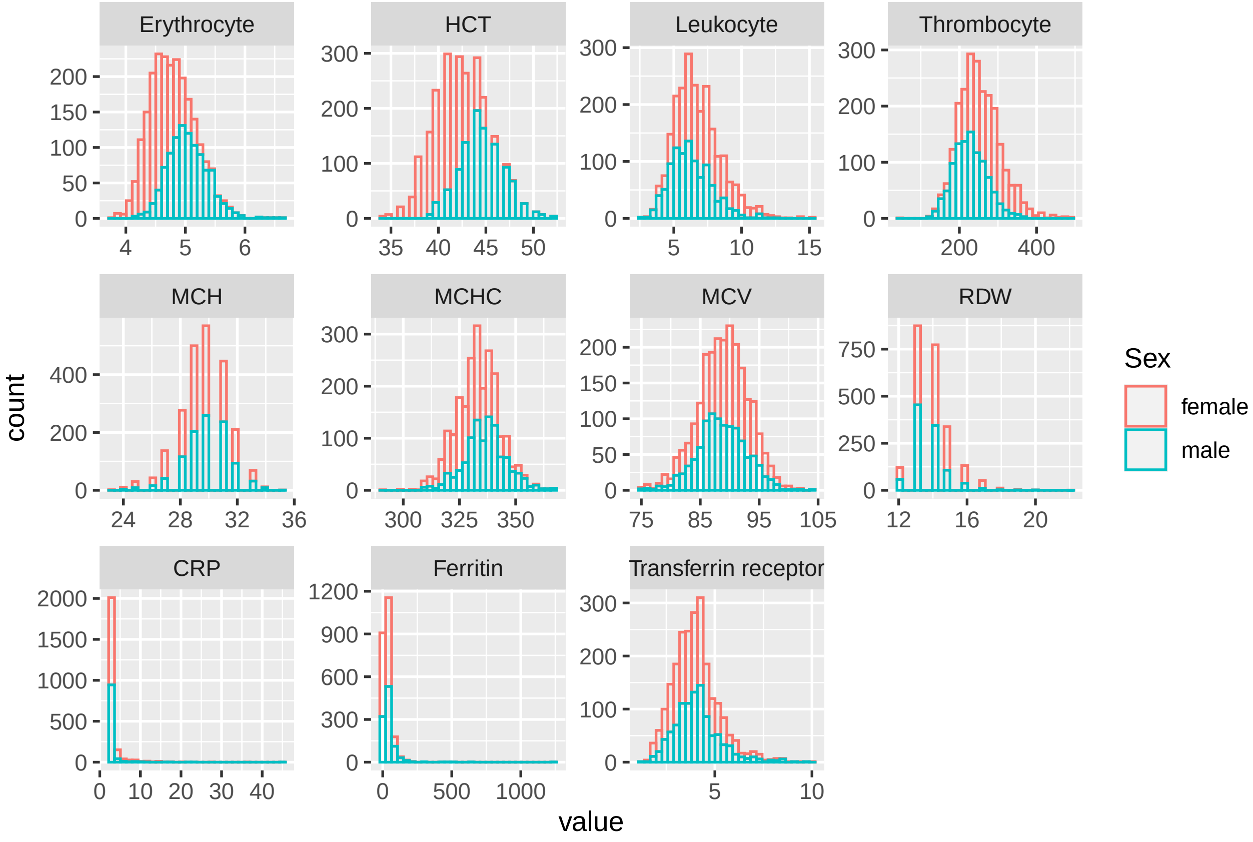

Figure S4: Summary plots of the eleven donation specific variables in the FinDonor data used in the linear models.

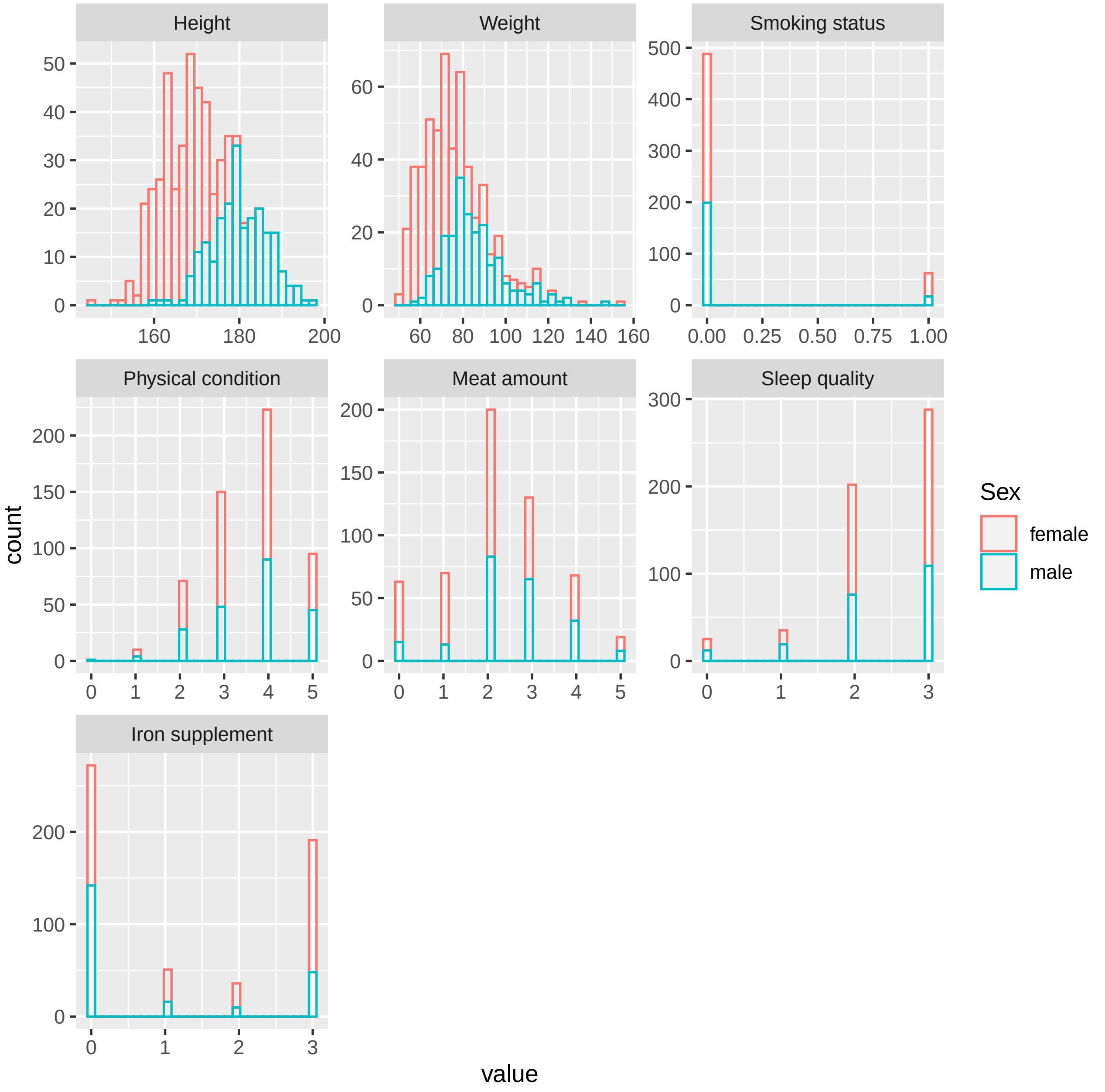

Figure S5: Summary plots of the seven donor specific variables in the FinDonor data used in the linear models. All the variables are self estimated. Physical condition - How would you rate your physical condition?: "very bad" = 0, "rather bad" = 1, "satisfactory" = 2, "rather good" = 3, "good" = 4, "excellent" = 5. Meat amount - How often do you eat meat?: "never" = 0, "less than once weekly" = 1, "1-3 times a week" = 2, "4-6 times a week" = 3, "daily" = 4, "several times daily" = 5. Sleep quality - How often do you feel like you've slept enoughy?: "never" = 0, "rarely" = 1, "most of the time" 2, "always or almost always" = 3. Smoking status - Do you smoke currently?: "no" = 0, "yes" = 1. Iron supplement - How many iron tablets offered to you after blood donation by FRCBS did you take?: "none or I was not offered any" = 0, "less than half" = 1, "about half" = 2, "all or almost all" = 3.

S2 Methods details

In this section we describe the statistical methods that we have used throughout this research and which are relevant for predicting Hb-values and low hemoglobin deferrals. First we describe the linear mixed model which we used to predict Hb-values. Then we describe the methodological framework which was used to fit the model and do predictions on new donors.

S2.1 Linear Mixed Model (LMM)

Linear mixed models (LMMs) are widely used for longitudinal data with grouping variables which in our case are blood donors. They have also been used in previous literature to predict hemoglobin values of blood donors [Fok18; Nas16]. The term mixed model refers to the fact that the model has both fixed and random effects. Fixed effects include the common variables which have been measured and random terms include donor-specific random intercepts.

The simplest linear mixed model is formulated as:

$$y_{it} = \mathbf{x_{it}}'\boldsymbol{\beta} + b_i + \varepsilon_{it} \tag{S1}$$

with

$$b_i \sim N(0, \sigma_b^2) \qquad \varepsilon_{it} \sim N(0, \sigma_\varepsilon^2).$$

The value y_{it} refers to the hemoglobin value of individual i at time t, vector $\mathbf{x_{it}}$ refers to explanatory variables of individual i at time t and $\boldsymbol{\beta}$ is the corresponding parameter vector. The random term b_i can be interpreted as average individual deviation from the population mean of Hb-levels. The individual deviation can be caused by external factors not described by the data such as genetic or dietary factors. For the consistency of parameter estimates it is assumed that the explanatory variables and random intercepts are independent. The error term ε_{it} is also assumed to be independent of the random intercept.

S2.1.1 Dynamic linear mixed model (DLMM) and the initial conditions problem

Usually we are interested in using the previous hemoglobin value as a predictor. For example if an individual is deferred from donation due to low hemoglobin, the next time he/she arrives to donate the hemoglobin can be much higher due to longer waiting period. We call a LMM with the previous hemoglobin value as a predictor a dynamic linear mixed model (DLMM). However, we can't treat the lagged response variable as any other predictor since this would violate the assumption that the correlation between the random intercept and the predictors is zero. This would be violated since the Hb-value at time $t = 0$ is endogenous (explained by other covariates). This is called the initial conditions problem. There are multiple solutions proposed to correct for these model violations in the statistical literature. We implemented two of these and will describe them here.

Heckman correction is a well known approach to fix the initial conditions problem [Hec81]. The idea is to jointly model an equation which links the random intercept of each individual to their first observations.

$$y_{i0} = \mathbf{z_i}'\boldsymbol{\upsilon} + b_i\theta + \eta_i \tag{S2}$$

where

$$\eta \sim N(0, \sigma_\eta^2).$$

Here $\mathbf{z_i}$ refers to exogenous variables that could be associated with initial observation for each donor. In our case exogenous variables are age, year, hour and warm_season. Vector $\boldsymbol{\upsilon}$ is corresponding parameter vector and η_i is the error term which is assumed to be independent of the random intercept. Parameter θ accounts for the possibility that the correlation between the first Hb-value y_{i0} and the random effect is non-zero.

Another approach for initial conditions problem is given by Wooldridge [Woo05]. The idea is to consider the distribution of the random effects conditional on the initial observation and exogenous variables. We formulate the distribution of the random effects as:

$$b_i = \zeta_0 + \zeta_1 y_{i0} + \mathbf{z_i}'\boldsymbol{\upsilon} + a_i \tag{S3}$$

Here $\mathbf{z_i}$ refers again to exogenous variables associated with the initial observation and a_i is the normally distributed error term which corresponds to the distribution of the random effects. Wooldridge correction is very easy to implement since we'll just set b_i in Equation S1 to be (S3). By doing this, a_i is our new random intercept term which is assumed to be uncorrelated with initial observation y_{i0} satisfying our model assumptions.

S2.1.2 Incorporating individual-specific variables

Some of the variables in our data had only one value for each donor. They are the Biobank variables in Table S2 and the questionnaire variables in Table S3. In order to add them as predictive variables to our model, we added additional parameter ϕ:

$$y_{it} = \mathbf{x_{it}}'\boldsymbol{\beta} + \mathbf{c_i}'\boldsymbol{\phi} + b_i + \varepsilon_{it}. \tag{S4}$$

S2.1.3 Bayesian framework

We did our modeling using Bayesian inference. For this kind of modeling Bayesian framework offers a lot of appealing qualities. We can incorporate our prior beliefs about variables distributions to the analysis in the form of *prior distributions*. Instead of getting our fitted coefficients as point estimates, we'll get them as *posterior distributions*. The same applies for predictions too.

We implemented our models and ran our analyses with Stan-platform [Car+17] which is state-of-the-art platform for statistical computation. It offers fully Bayesian inference with MCMC sampling. Our models were fit mainly using `Rstan` and occasionally with `CmdStan` in case of more memory-heavy models. The Stan-models are shared in our GitHub repository and Stan-files are described in Tables S4 and S5.

The exact sampling algorithm that was used for Markov chain Monte Carlo sampling was the Stan's default No-U-Turn Hamiltonian Monte Carlo sampler (NUTS). Hamiltonian Monte Carlo avoids some of the sampling issues that simpler methods such as Gibbs Sampler suffer from. These include random walk behaviour and sensitivity to correlated parameters. This allows HMC to converge to high-dimensional target distributions more quickly than standard methods. The No-U-turn variant of HMC eliminates the need to set proper user-specified parameters which could lead into large waste of computation if chosen incorrectly [HG11].

S2.1.4 Prior specifications

We used conjugate priors for all of our parameters for more straight-forward inference. By using conjugate priors our posteriors will be of known form. For the variance parameters for random effects and residuals σ_b^2 and σ_ε^2 we used inverse gamma priors and for other parameters we consider a normal prior. We also took into account the prior choice recommendations of the Stan-developers. These priors can be thought of generic weakly informative priors.

$$\sigma_b^2 \sim IG(0.01, 0.01), \qquad \sigma_\varepsilon^2 \sim IG(0.01, 0.01) \qquad \beta_i \sim N(0,1),\ \forall i \in 1, \ldots, K \tag{S5}$$

where K is the number of predictor variables. The donor-specific variables have the following prior distributions:

$$\phi_i \sim N(0,1),\ \forall i \in 1, \ldots, M, \tag{S6}$$

where M is the number of donor-specific variables.

The Heckman correction jointly models the initial response of every individual. This brings a need to specify priors for parameters in Equation S2. Once again we use conjugate priors for the parameters, using inverse gamma for variance and normal for other parameters:

$$\upsilon_i \sim N(0,1),\ \forall i \in 1, \ldots, L, \qquad \sigma_\eta^2 \sim IG(0.01, 0.01), \qquad \theta \sim N(0,1) \tag{S7}$$

where L is the number of exogenous variables at initial event.

For Wooldridge correction we also need priors for additional parameters. We use conjugate priors for parameters in Equation S3. Just like other parameters we set inverse gamma prior for variance and normal prior for other parameters:

$$\zeta_0, \zeta_1 \sim N(0,1), \qquad \upsilon_j \sim N(0,1),\ \forall j \in 1, \ldots, L, \qquad a_i \sim N(0, \sigma_a^2), \qquad \sigma_a \sim IG(0.01, 0.01). \tag{S8}$$

S2.1.5 Out-of-sample predictions

In order to properly evaluate the performance of our models one would need to do predictions outside of the training data. First the whole data set is split into a training set and a validation set. The training set is used to fit the model parameters and the validation set is used to compare how well the model is able to predict Hb-values for donors that were not in the training sample. The general predictive likelihood of observations in the validation set is given by:

$$p(\mathbf{y}_{test}|\mathbf{y}_{train}, \mathbf{X}) = \int p(\mathbf{y}_{test}|\boldsymbol{\theta}, \mathbf{X}_{test})p(\boldsymbol{\theta}|\mathbf{X}_{train}, \mathbf{y}_{train})d\boldsymbol{\theta}, \tag{S9}$$

where parameter vector $\boldsymbol{\theta}$ contains all fixed model parameters. To obtain point estimates of the predictions we use the mean value of posterior predictive distribution $p(\mathbf{y}_{test}|\mathbf{y}_{train}, \mathbf{X})$. We used a sample size of 1000 draws from the posterior distributions for all parameters but this is a user-defined parameter in our prediction functions which can be found in `final_models.Rmd` file. Since we are using mixed models we can not simply use only the parameter vector $\boldsymbol{\theta}$ to do the predictions, since we also need to find out the random effects b_i of the previously unseen donors. However, we can describe the posterior distribution of the donor specific intercept analytically and sample from that, as described in the next subsection.

We used 4-fold cross-validation to validate our FinDonor models, to see whether the models are overfitting. In this workflow we first split the data into four equal-sized random sets of donors and fit the model four times using in turn one set as a validation set and the other sets combined as the training set. We then average the validation results of the different choices of the validation set to get the average performance.

S2.1.6 Dynamic predictions

The Hb-predictions of an individual are based on the fixed model parameters, individual covariate values and the donation history. We can estimate the random intercept of each individual using this known information. After computing the posterior of the fixed parameter vector $\boldsymbol{\theta}$ we can dynamically estimate the random intercept of each individual at each time point in his/her donation history. The posterior distribution of the random intercept is given by:

$$p(b_{it}|y_{i1}, ..., y_{it-1}\mathbf{X}_i, \boldsymbol{\theta}) \propto p(y_{i1}, ..., y_{it-1}|\mathbf{X}_i, b_{it}, \boldsymbol{\theta})p(\mathbf{b}), \tag{S10}$$

where b_{it} is the random intercept of individual i at time t. The subscript t is used here since b is a dynamic parameter dependent of time. To estimate b_{it} at time t we can use the past information available until time $t-1$ for individual i. This also means that we can update b_{it} for all events in individual's donation history and that we can update the intercept term whenever we get new information. The conditional posterior in Equation S9 is normal distribution since the model is linear in the random effects:

$$p(b_{it}|\mathbf{X}_i, \boldsymbol{\theta}, y_{i1}, ..., y_{it-1}) = N(\bar{b}_i, \sigma^2_{b_i}), \tag{S11}$$

where $\bar{b}_i$ is the posterior mean of the random intercept and $\sigma^2_{b_i}$ is the posterior variance.

We used the same method as [Fok18] to find close form expression for the posterior mean and variance for the Heckman correction. First, from Equation S2 of the initial observation we get

$$\frac{y_{i0} - \mathbf{z}_i'\boldsymbol{\upsilon}}{\sigma_\eta} = \frac{b_i\theta}{\sigma_\eta} + \frac{\eta_i}{\sigma_{\eta_i}}. \tag{S12}$$

We do the same for subsequent observations of individual i:

$$\frac{y_{it} - \mathbf{x}_{it}'\boldsymbol{\beta}}{\sigma_\varepsilon} = \frac{b_i}{\sigma_\varepsilon} + \frac{\varepsilon_{it}}{\sigma_\varepsilon}. \tag{S13}$$

To formulate the sampling distribution of the random intercept of individual i at time $t+1$ we can stack the observations at times $0, ..., t$ for every individual, resulting in two new variables:

$$\mathbf{y}_i^* = \begin{pmatrix} (y_{i0} - \mathbf{z}_i'\boldsymbol{\upsilon})/\sigma_\eta \\ (y_{i1} - \mathbf{x}_{i1}'\boldsymbol{\beta})/\sigma_\varepsilon \\ \vdots \\ (y_{it} - \mathbf{x}_{it}'\boldsymbol{\beta})/\sigma_\varepsilon \end{pmatrix}, \qquad \mathbf{x}_i^* = \begin{pmatrix} \theta/\sigma_\eta \\ 1/\sigma_\varepsilon \\ \vdots \\ 1/\sigma_\varepsilon \end{pmatrix}. \tag{S14}$$

For a fixed individual i, b_i is a parameter of an ordinary linear regression model with standard normally distributed error term. Since the prior $p(\mathbf{b})$ of random intercepts was defined as a normal distribution we can use the conjugacy to sample the random intercept b_i of individual i at time $t+1$ from a normal $N(m^*, s^*)$, where:

$$m^* = s^* \mathbf{x}_i^{*\prime} \mathbf{y}_i^* \qquad \text{and} \qquad s^* = \left(\mathbf{x}_i^{*\prime} \mathbf{x}_i^* + \sigma_b^{-2}\right)^{-1}. \tag{S15}$$

In order to estimate the value of b_i at time $t+1$ we can only use observations of individual i up to time t.

To sample the random intercept term for the LMM shown in Equation S1, which does not use the lagged response as a predictor, we can simply apply the same method. Since we don't handle the initial response differently we can treat all observations as in Equation S13. By stacking the observations we get auxiliary variables:

$$\mathbf{y}_i^* = \begin{pmatrix} (y_{i1} - \mathbf{x}_{i1}'\boldsymbol{\beta})/\sigma_\varepsilon \\ \vdots \\ (y_{it} - \mathbf{x}_{it}'\boldsymbol{\beta})/\sigma_\varepsilon \end{pmatrix}, \qquad \mathbf{x}_i^* = \begin{pmatrix} 1/\sigma_\varepsilon \\ \vdots \\ 1/\sigma_\varepsilon \end{pmatrix}. \tag{S16}$$

We can sample from the $N(m^*, s^*)$ distribution as shown above. Here it is worth mentioning that the indexing of time in (S16) starts from 1 due to the fact that we use the first Hb-value as a predictor for all events, so we remove the first events for all individuals from the training data.

S2.2 Random forest

Random forest is a non-linear ensemble model for classification [Bre01]. It basically constructs a multitude of decision trees, applies them to data and reports the mode, that is the most common classification, predicted by the decision trees. Individual decision trees are known to be unstable and prone to overfitting [Tan+05; Bre01]. An individual tree is built by finding a single variable that best splits the data in two in relation to the class that is being predicted. Then the same process is repeated for each of the two groups created by previous split and so on until no improvements in the classification is detected. We use the R-library `rpart` [TA19] to fit decision tree and `randomForest` [LW02] to fit a random forests. N.B. the linear mixed model approaches above try to predict the actual hemoglobin concentration while here we use random forest for classification i.e. to directly predict if donor will be deferred or not.

To create a balanced set of cases (deferrals) and controls (whole blood donations), required commonly by machine learning methods, we oversample the timeseries of donors who have deferral as their last donation. With random forest this oversampling is performed once per each tree in the forest to create a separate data from which to train the tree. We then use only the last event and all the variables derived for it (Table S1) as the data set. Also, as the full time series data is excluded, we add a variable for total number of donations by the donor i.e. "Life time donations".

The hyperparameter tuning is done with 5 times repeated 4-fold cross-validation i.e. by splitting randomly the data into four parts, using three parts to train the model and one part to validate and repeating this 5 times. This is carried out for each hyperparameter value combination to find the best combination i.e. we carry out a grid search using functionalities of the `caret` library [Kuh20]. We then fit a single decision tree with default settings of the `rpart` library as a baseline and example. For the decision tree the only hyperparameter was `cp`, which limits the complexity of the tree. For random forest we tune with our train set one hyperparameter, `mtry`, which gives the number of variables that are randomly tested in each split. For both the decision tree and random forest we first fit the model with a training set (64% random sample of data set), then evaluate the performance of the model with validation set (16% of data) and then finally at the end of the project when all modelling choices have been made test model performance with a test set(20% of data).

S2.3 Model implementations

We have created an easy-to-use user interface to the hemoglobin predictors that works in any web browser. As our implementation requires numerous R packages (about 20) and other software, which can be tedious to install, we have created a software container that includes all our implementations and their dependencies in one package for easy installation. The software container can be run on any machine (Linux, Windows, or macOS) that has `docker` [Inc20] installed. The ready-to-use binary version of the software container for hemoglobin prediction is available currently from DockerHub (`https://hub.docker.com/r/toivoja/hb-predictor`).

We have made publicly available all the source code necessary to perform the same analyses and visualizations as we have done in this report. The source code can be found in GitHub:

Hb_predictor_container: Code for hemoglobin and deferral prediction using LMMs and random forest models implemented in R and Stan. In addition, code for creating the software container that includes an easy to use user interface to the predictors working inside a web browser, and all the supporting code and their dependencies is provided. Also, code to generate most of the figures and tables in the article and this supplement is available. (`https://github.com/FRCBS/Hb_predictor_container`)

As previously mentioned we implemented our linear mixed models in a probabilistic programming language Stan. The Stan models that are provided in our GitHub repository are described in Tables S4 and S5. An example Stan code that computes hemoglobin predictions and samples from the posterior distribution using the LMM of Equation S1 is shown in Listing 1.

Code file	Model description
`container.stan`	LMM as in Equation S1.<br>Uses initial Hb instead of lagged Hb as a predictor.
`container_consts.stan`	Same as above with added donor specific variables as in Equation S4.
`container_heckman.stan`	DLMM as in Equations S1 and S2.<br>Uses lagged Hb as a predictor.
`container_heckman_consts.stan`	Same as above with added donor specific variables as in Equation S4.

Table S4: Linear mixed models written in Stan for in-sample-prediction and their short descriptions.

Code file	Model description
`oos.stan`	LMM as in Equation S1.<br>Uses initial Hb instead of lagged Hb as a predictor.
`oos_consts.stan`	Same as above with added donor specific variables as in Equation S4.
`oos_heckman.stan`	DLMM as in Equations S1 and S2.<br>Uses lagged Hb as a predictor.
`oos_heckman_oos.stan`	Same as above with added donor specific variables as in Equation S4.

Table S5: Linear mixed models written in Stan for out-of-sample prediction and their short descriptions.

S2.4 Performance metrics

To estimate the performance of our models we use multiple widely used error estimates to evaluate how well our models can perform predictions. We use root mean squared error (RMSE) and mean absolute error (MAE). We also use our predicted values to evaluate the eligibility of blood donors. In Finland the Hb-values are measured in grams per litre [g/L]. If the Hb-value of a donor is under the deferral threshold, the donor will be deferred from donating due to low hemoglobin. These deferral thresholds are 135 g/L for male donors and 125 g/L for female donors. To assess the performance of our models in identifying low hemoglobin deferrals we compute the receiver operating characteristic (ROC) curve. We used the fraction of posterior draws falling under deferral threshold and compared it to the true label to create the ROC-curve. We then calculate the area under the curve (AUROC) and use this to compare the models. The AUROC value can be thought of a measure of discrimination, or in other words the ability of a model to correctly identify individuals who are going to be deferred. Since the data set is very imbalanced, the fraction of deferrals among donations is only 4%, we compute and show the precision recall curve (PR) and the area under it (AUPR). AUPR could give a more reliable measure of classifier performance in this case. Note that, since we are trying to predict whether a donation is deferred or not, in our terminology the deferral is the positive class and acceptance is the negative class. In the ROC and PR plots we also show the 95% confidence intervals for the AUROC and AUPR values, which are computed using the `basic` method of the `boot` R package [CR20] with as many repetition as there are data points, but at least 10 000 repetitions. We admit that the estimated intervals may be slightly biased in some cases, but the memory usage of the bias-corrected and accelerated method (BCa) [TE93] was prohibitive for our larger data sets.

Listing 1: Example Stan code of the LMM.

```
// Author: Yrjö Koski, 2020
// Modified by: Jarkko Toivonen, 2020

data {
    // Train data
    int<lower=1> N;             // Number of donation events
    int<lower=1> K;             // Number of variables measured at each donation
    int<lower=1> N_donor;       // Number of donors in our data
    matrix[N, K] X;             // Data matrix X
    vector[N] Hb;               // Hemoglobin values (that we are trying to predict)
    int<lower=1, upper=N_donor> donor[N];               // Donor identifier

    // Test data
    int<lower=1> N_test;        // Number of test observations
    matrix[N_test, K] X_test; // Test data
    int<lower = 1, upper=N_donor> test_donor[N_test]; // Test donor identifier
}
parameters {
    vector[K] beta;             // Coefficients on X
    real<lower=0> sigma_b;      // Variance of the individual intercept b
    real<lower=0> sigma_eps;    // Variance of the random noise
    vector[N_donor] donor_b;    // Donor specific random effect
}
model {
    // A priori distributions
    beta        ~ std_normal();
    sigma_b     ~ inv_gamma(0.01, 0.01);
    sigma_eps   ~ inv_gamma(0.01, 0.01);
    donor_b     ~ normal(0,sigma_b);

    // Distribution for hemoglobin
    Hb          ~ normal(X * beta + donor_b[donor], sigma_eps);
}
generated quantities {
    vector[N_test] y_pred;

    // Predict using parameters and test data
    for (i in 1:Ntest) {
        y_pred[i] = normal_rng(X_test[i]*beta + donor_b[test_donor[i]], sigma_eps);
    }
}
```

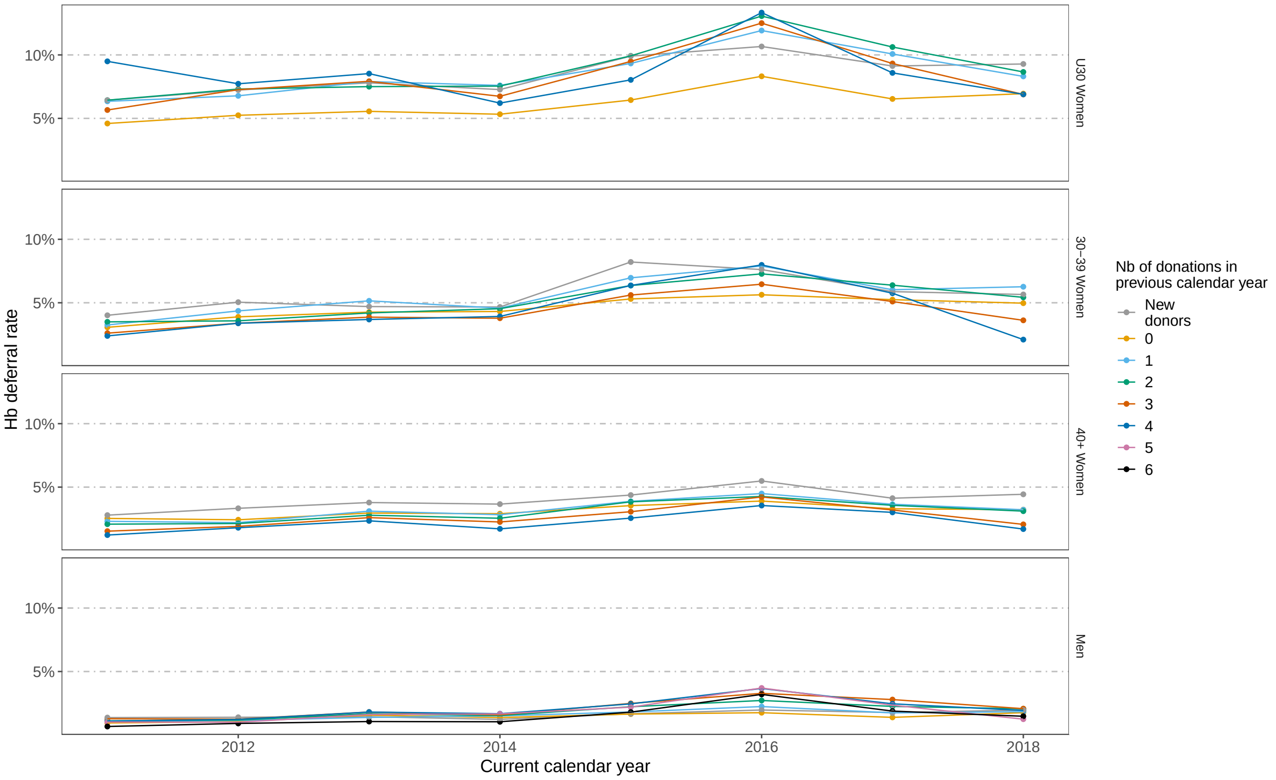

Figure S6: Effect of previous donation activity on deferral rate. On x-axes year and y-axes low hemoglobin deferral rate. The rates are divided into number of donations on previous year (colors) and demographic groups (separate plots). The rise in deferrals in year 2016 was likely caused by batch-to-batch variation of capillary hemoglobin testing cuvettes [Cas+18].

S3 Results details

S3.1 Definition of alternative donation intervals

An initial task of the project was to pre-define alternative donation intervals based on available data. These would be required in case a computational predictor could predict deferral events, but it could not exactly predict when should a donor return for a successful blood donation. The alternative donation intervals could then be used to estimate potential economic effects of deferral predictors. To this end we explored donation history data from the FRCBS eProgesa data base and the FinDonor data.

We first investigated the proportion of deferred donations in years 2011–2018 in eProgesa data. The effect of previous donation activity (here computed as the number of donations during the previous calendar year) on low hemoglobin deferral rates in the current calendar year varies as a function of donor demographic groups (Figure S6). Low hemoglobin deferral rates were computed across all donations and thus represent the percentage of total donation attempts that were low hemoglobin deferrals (not to be confused with the percentage of donors who are deferred). Overall, the percentage of deferred donations was higher in younger women than in older women and lower in men than in women. In women but not in men, the percentage of deferred donations tended to be higher for donations from new donors than for donations from repeat donors. In women aged 30 and over, the percentage of deferred donations was lowest for highly active donors. In women aged less than 30 in all years, except year 2018, the deferral rate was lowest for repeat donors who had not donated in the previous calendar year. Based on this result decreasing the allowed number of yearly donations for most demographic groups will not result in a decrease of the group's deferral rate. However, giving to a donor that is predicted to be deferred, a donation interval of one donation per year or a deferral lasting a year is likely to very effectively lower their chances of subsequent deferral. In particular this is true for women aged less than 30 where most deferrals take place.

The finger-prick capillary hemoglobin measurements presented above represent the whole blood donor population in Finland. However, capillary hemoglobin has much higher error than venous measurements [Bäc+20]

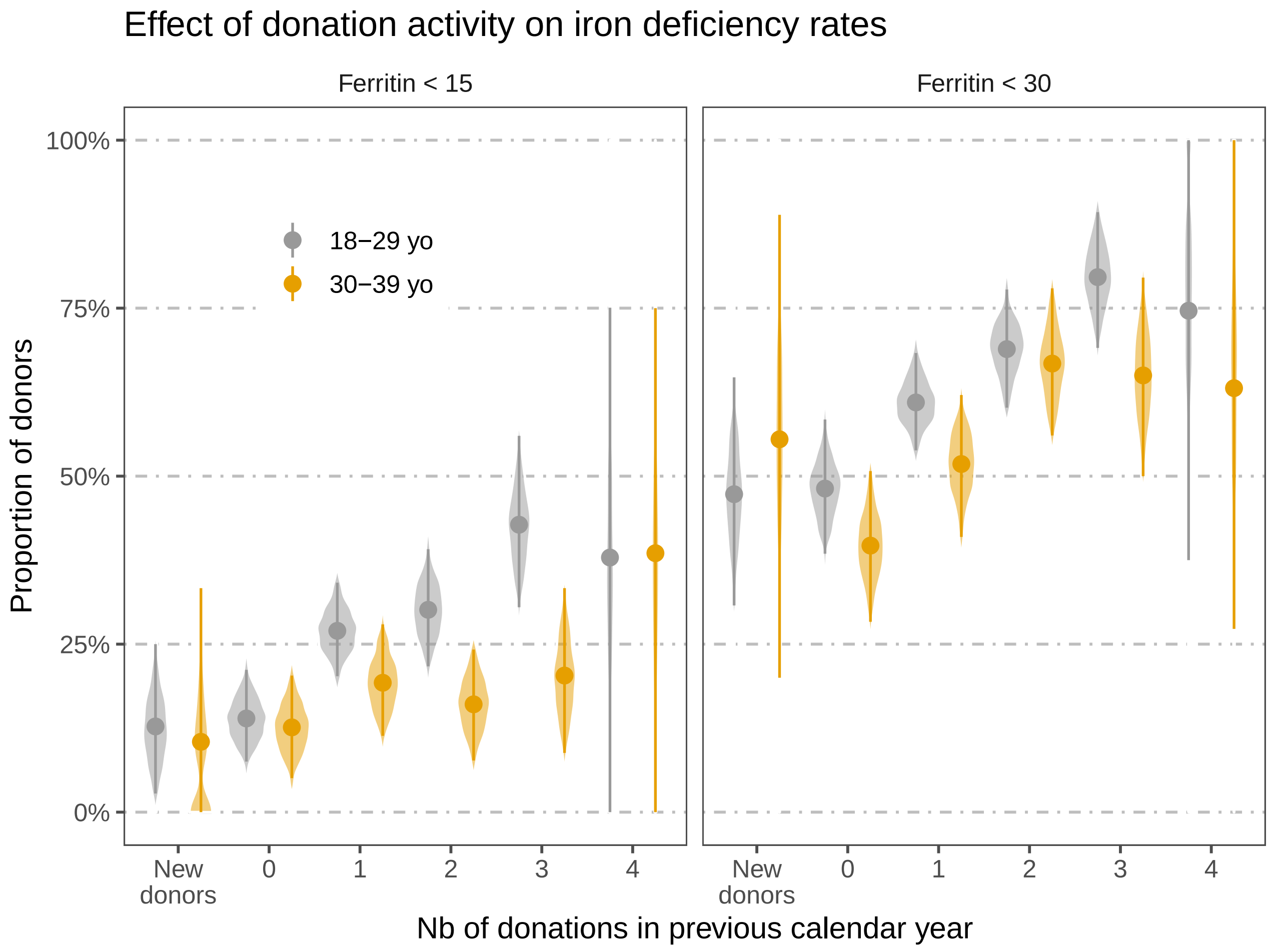

Figure S7: Bootstrapped effect of donation activity on iron deficiency rates of women. Point estimate of the prevalence (dot), 95% confidence interval of the prevalence (vertical line) and the bootstrapped distribution of the prevalence (background around the vertical line) are shown.

and in general hemoglobin correlates weakly with iron stores [Lob+19]. Hence, it could inform us poorly on how long donation delays are required for recuperation. We therefore use data from the FinDonor project to explore the effect of donation frequency on ferritin levels. With approximately 2500 participants, the FinDonor cohort is only a small sample of the actual donor population. To alleviate this source of error we use bootstrapping to estimate descriptive statistics from the data. That is, we pick with replacement 1000 times a random subset of the data of equal size as the original data. All statistics are then computed across these 1000 samples. We compute the prevalence of iron deficiency (Ferritin $< 15\,\mu g/L$) and low iron (Ferritin $< 30\,\mu g/L$) as a function of donation activity and age group (Figure S7). The confidence intervals are very large for new young female donors and donors with high donation frequency indicating that we do not have enough data for them. The proportions of low iron donors with zero or one donation in the previous calendar year appear similar, while for those with three or four donations in the previous year the proportion of low iron appears markedly higher. A similar but weaker pattern appears in the iron deficiency proportions. We interpret this result as the previous result on hemoglobin deferral rate i.e. deferring blood donation for a year is likely to allow iron level recuperation very efficiently even for donors in risk groups.

In our data high donation activity appears to be correlated with low iron stores (Figure S7), but not necessarily with high deferral rates (Figure S6). This is likely a result of self selection of donors towards those who tolerate blood donation well. The FRCBS blood service provides iron supplementation for risk group donors. It has been shown that rigorous adherence to low dose iron supplementation allows hemoglobin recovery to predonation level in a month and ferritin recovery in less than five months [Kis+15]. Also, FRCBS medical doctors routinely assign half year deferrals for donors based on clinical assessment and low pre-donation hemoglobin. This has been found to be in practice very effective for allowing the hemoglobin to recover. Hence,

we choose to analyse also the effect of six month deferral or donation interval, in addition to the 12 month, in our estimation of economic effects of deferral predictors.

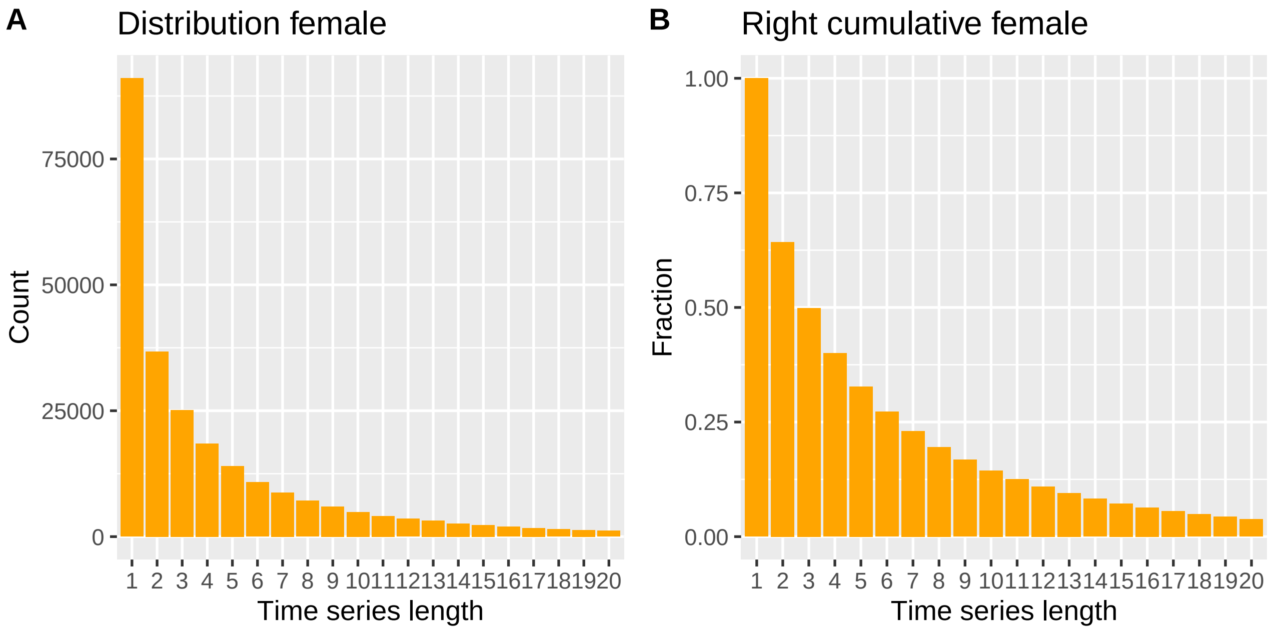

Figure S8: The distribution of time series length of female donors in eProgesa data set. (A) The frequencies of the time series lengths. (B) The right cumulative distribution of the time series length, that is, the fraction of donors having at least the shown time series length.

Table S6: The effect of time series length on hemoglobin prediction error and deferral classification performance. DLMMs were fitted on female eProgesa data. Only the donors with the wanted number of donations (column Length) were considered in turn. The results seem to improve as the time series length increases.

	g/L		mmol/L			
**Length**	**MAE**	**RMSE**	**MAE**	**RMSE**	**AUROC**	**AUPR**
4	7.68	12.57	0.47	0.78	0.77	0.23
5	6.91	8.90	0.43	0.55	0.81	0.27
6	6.90	8.86	0.43	0.55	0.80	0.24
7	6.79	8.74	0.42	0.54	0.82	0.26
8	6.65	8.53	0.41	0.53	0.80	0.20
9	6.75	8.66	0.42	0.54	0.81	0.27
10	6.77	8.65	0.42	0.54	0.83	0.24

S3.2 Development of computational predictors

S3.2.1 Exploring data

Table S7: The effect of the number of donors on prediction. DLMM was fitted for each random subset of female eProgesa data, with the shown numbers of donors. No restriction on the time series length was applied.

	g/L		mmol/L			
**Sample size**	**MAE**	**RMSE**	**MAE**	**RMSE**	**AUROC**	**AUPR**
10 000	6.93	8.91	0.43	0.55	0.80	0.23
30 000	6.95	8.96	0.43	0.56	0.77	0.22
50 000	6.98	8.99	0.43	0.56	0.79	0.23

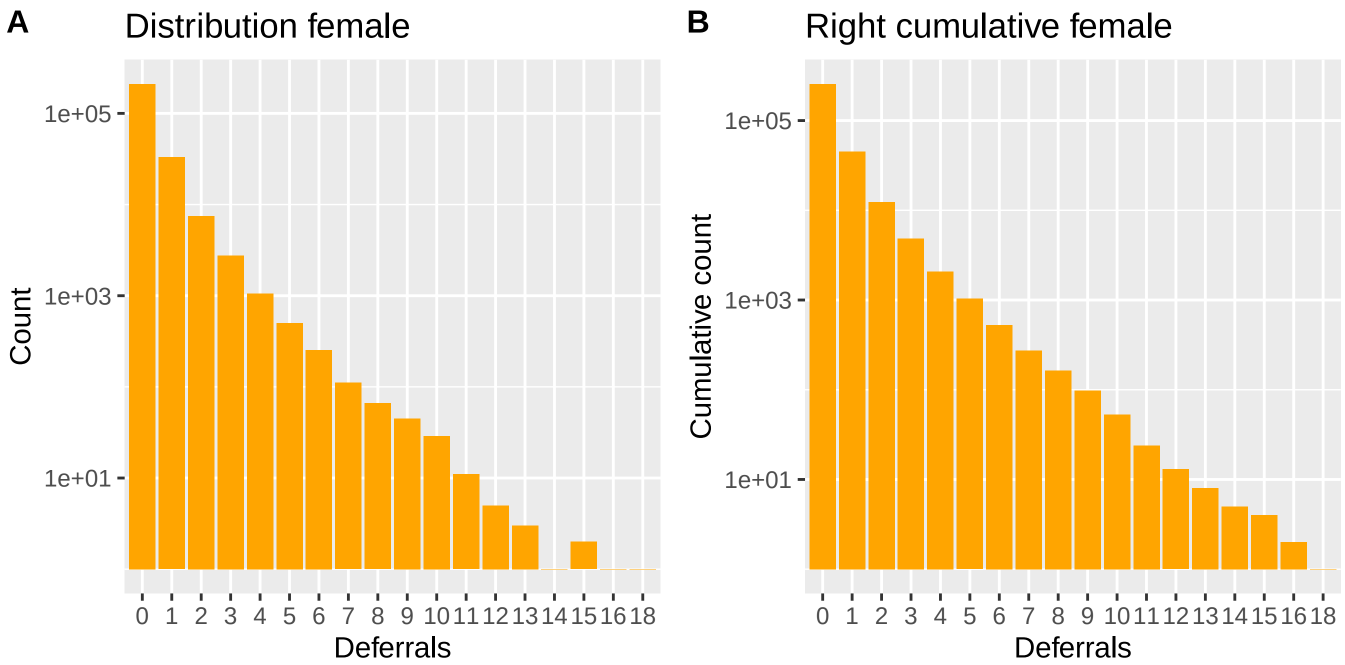

Figure S9: The frequencies of deferrals per donor in female eProgesa data. (A) The frequencies of each deferral count. (B) The right cumulative count of each deferral count, that is, the number of donors with at least the shown number of deferrals. The y-axes are in logarithmic scale, since the number of donors drops rapidly as the function of deferrals per donor.

S3.2.2 Linear mixed models on eProgesa data

We fitted two different linear models for the test half of the eProgesa data set. The models are:
1. LMM with initial Hb-value as a predictor, and
2. DLMM with lagged Hb-value as a predictor.

We ran both of these models separately for male and female donors. In all the experiments only donors with at least seven donations were considered. The variables used in the models are described in Table S1. We used Heckman correction for all DLMMs.

The effect sizes of variables on hemoglobin values are visualized in Figures S10–S11 and shown in numeric form in Tables S9 and S10. In addition to the posterior mean, the 95% highest posterior density interval (HPDI) is shown as well for each variable. The biggest difference between sexes seems to be in the age variables: in females the age and first age variables have quite large positive effect whereas for males the effect is smaller and negative. In LMMs the first hemoglobin value seems to have the largest effect, and in DLMMs the previous hemoglobin value has the largest effect. This is to be expected as the LMMs do not use the previous hemoglobin value. The confidence intervals are quite narrow (except for the previous hemoglobin deferral), due to the large size of eProgesa data.

The Figures S12–S15 show the observed and predicted hemoglobin values as a scatter plot and the observed and predicted deferrals as a confusion matrix. The mean of the posterior distribution for hemoglobin is used as the point estimate. In the scatter plots a generalized additive model (GAM) is fitted to the points, and

Table S8: The effect of enrichment of deferrals in female eProgesa data. After the enrichment 50% of the donors had at least one deferral. We fitted a DLMM on this data and on an unenriched sample of 30 000 donors from female eProgesa data, where 12% of the donors had at least one deferral. The prediction errors and deferral classifier got worse after the enrichment.

		g/L		mmol/L			
**Donors**	**Enrichment**	**MAE**	**RMSE**	**MAE**	**RMSE**	**AUROC**	**AUPR**
30 000	0.12	6.95	8.96	0.43	0.56	0.79	0.22
37 162	0.50	8.20	10.50	0.51	0.65	0.68	0.34

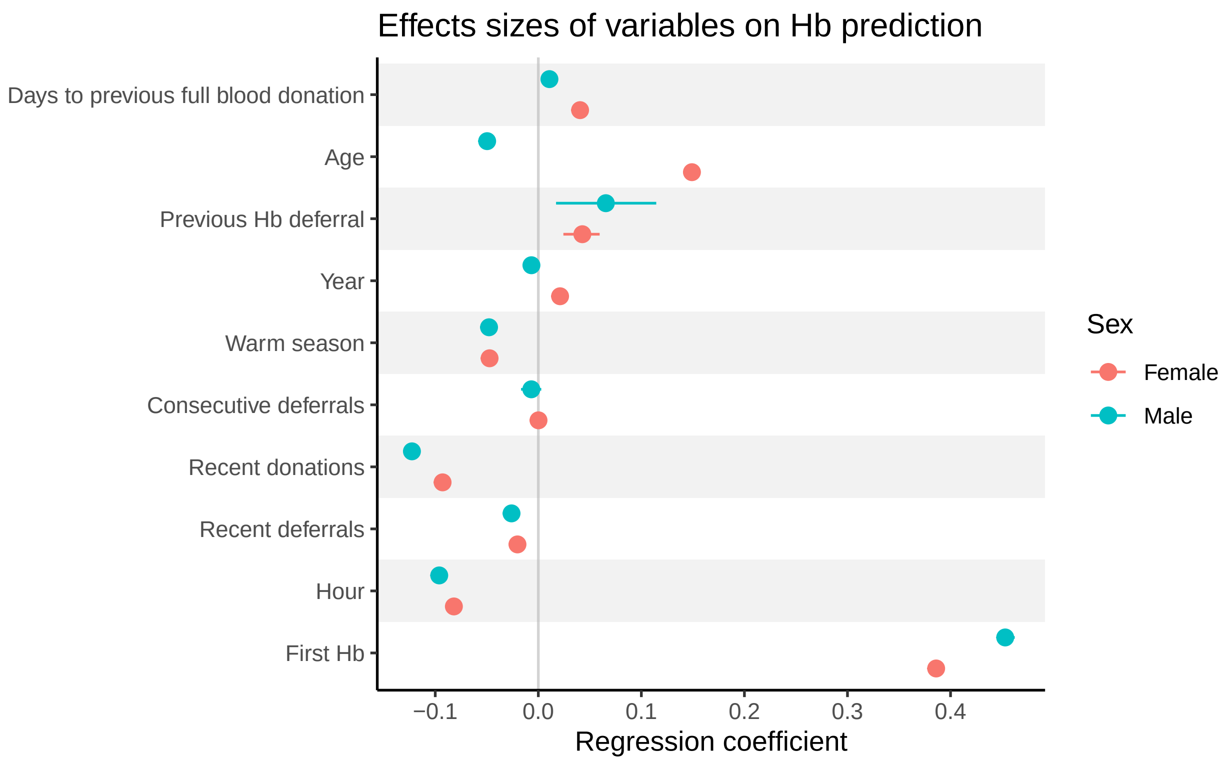

Figure S10: The effect sizes of the variables used in predicting hemoglobin with a LMM on eProgesa data. The predicted hemoglobin is the weighted sum of the predictor values and the corresponding coefficients learned by the fitting algorithm. These coefficients, or effect sizes, of the variables tell how large an effect each variable has on the predicted hemoglobin value. For example, in this model the first hemoglobin value has the largest effect on the predicted hemoglobin value, and this effect is positive. In addition to the mean of the posterior distribution the 95% highest posterior density interval (HPDI) is shown for each variable. There is 95% probability that the coefficient is contained in this interval. This credible interval (HPDI) has the additional property that it is most narrow of such credible intervals.

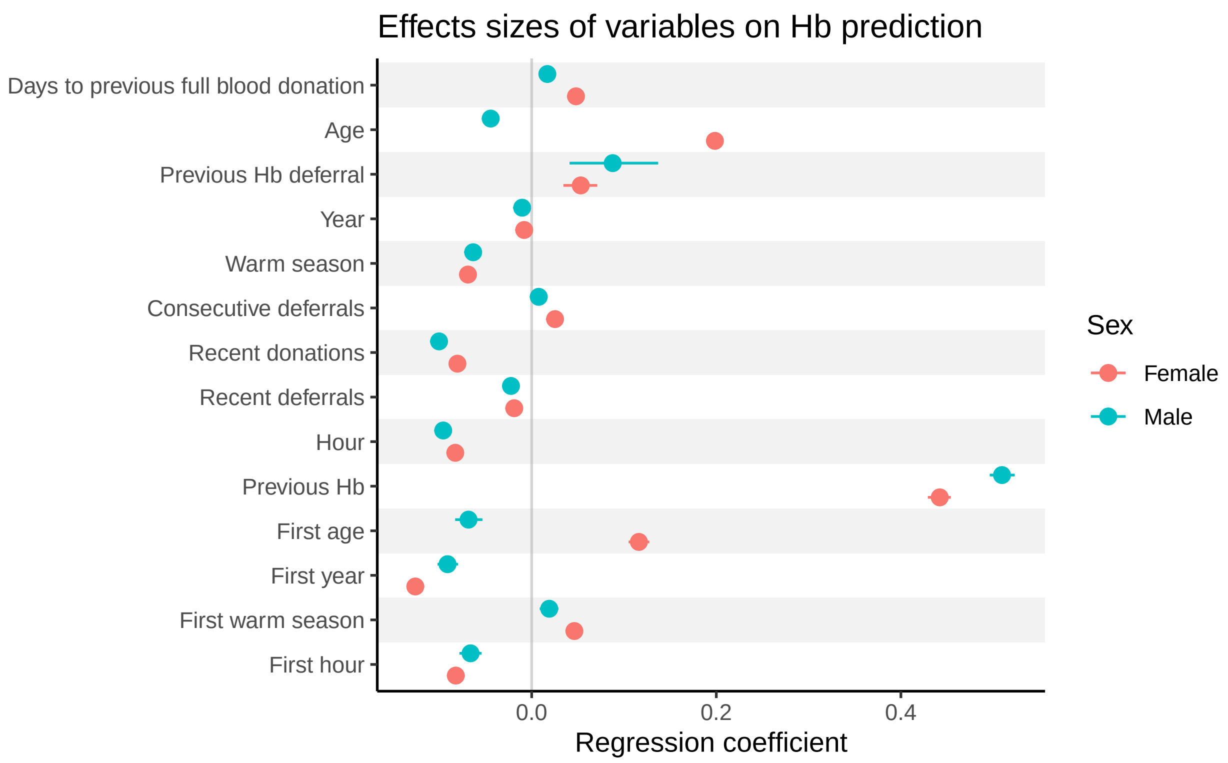

Figure S11: The effect sizes of the variables used in predicting hemoglobin with a DLMM on the eProgesa data. In addition to the mean of the posterior distribution the 95% highest posterior density interval (HPDI) is shown for each variable. See Figure S10 for details.

Table S9: The effect sizes of the variables used in predicting hemoglobin with a LMM on eProgesa data. In addition to the mean of the posterior distribution the 95% highest posterior density interval (HPDI) is shown.

	**Female**			**Male**		
	**mean**	**95% CI**		**mean**	**95% CI**	
Days to previous full blood donation	0.041	0.038	0.044	0.011	0.008	0.014
Age	0.149	0.143	0.156	-0.050	-0.057	-0.041
Previous Hb deferral	0.043	0.024	0.060	0.066	0.017	0.114
Year	0.021	0.017	0.025	-0.007	-0.011	-0.002
Warm season	-0.047	-0.050	-0.045	-0.048	-0.050	-0.045
Consecutive deferrals	0.000	-0.007	0.008	-0.007	-0.017	0.003
Recent donations	-0.093	-0.096	-0.090	-0.123	-0.127	-0.118
Recent deferrals	-0.020	-0.024	-0.016	-0.026	-0.030	-0.022
Hour	-0.082	-0.085	-0.079	-0.096	-0.099	-0.093
First Hb	0.386	0.380	0.393	0.453	0.445	0.462

Table S10: The effect sizes of the variables used in predicting hemoglobin with a DLMM on eProgesa data. In addition to the mean of the posterior distribution the 95% highest posterior density interval (HPDI) is shown.

	**Female**			**Male**		
	**mean**	**95% CI**		**mean**	**95% CI**	
Days to previous full blood donation	0.048	0.045	0.051	0.017	0.014	0.020
Age	0.199	0.191	0.205	-0.044	-0.053	-0.035
Previous Hb deferral	0.053	0.034	0.071	0.088	0.041	0.137
Year	-0.008	-0.012	-0.004	-0.010	-0.015	-0.005
Warm season	-0.069	-0.071	-0.066	-0.063	-0.066	-0.061
Consecutive deferrals	0.025	0.018	0.032	0.008	-0.002	0.017
Recent donations	-0.080	-0.084	-0.077	-0.100	-0.105	-0.096
Recent deferrals	-0.019	-0.022	-0.015	-0.022	-0.026	-0.019
Hour	-0.083	-0.086	-0.080	-0.096	-0.099	-0.093
Previous Hb	0.442	0.429	0.454	0.510	0.496	0.523
First age	0.116	0.105	0.127	-0.068	-0.083	-0.053
First year	-0.126	-0.134	-0.116	-0.091	-0.102	-0.080
First warm season	0.046	0.039	0.054	0.019	0.009	0.029
First hour	-0.082	-0.091	-0.073	-0.066	-0.078	-0.054

additionally the 95% confidence intervals are shown (in gray). The fitted GAM curve shows that the model has difficulties in predicting the lower hemoglobin values, which are exactly the ones we are interested in! The imbalance between classes and the large number of false negatives is clearly visible in the confusion matrices.

The performance of the binary classifier, based on dichotomising the predicted hemoglobin level, are depicted in Figures S16–S19. Both the receiver operating characteristic (ROC) and the precision-recall (PR) curves as well as the areas under the curves are shown. The ROC curves are shown for comparison with previous literature, but they give overly optimistic picture of the situation due to the imbalance of the classes. For this reason the precision-recall curves are shown as well as they are thought to better take into account the imbalance.

An optimal predictor could predict the number of days when the donor's hemoglobin has recuperated. The 95% credible intervals of "Days to previous full blood donation" do not cross zero in DLMM (Table S10) nor in LMM (Table S9) model and the coefficient is positive. Hence, we tested could the model predict recuperation of hemoglobin. This was done by creating test data where all the time dependent variables (all except first and previous hemoglobin and 'Previous Hb deferral') were updated as to simulate that the donor would arrive later than she actually did. Then the model was used to simulate a new hemoglobin value. This process was repeated by extending the event a month further consequently. However, no hemoglobin recuperation could be predicted by the model in any time frame of practical relevance.

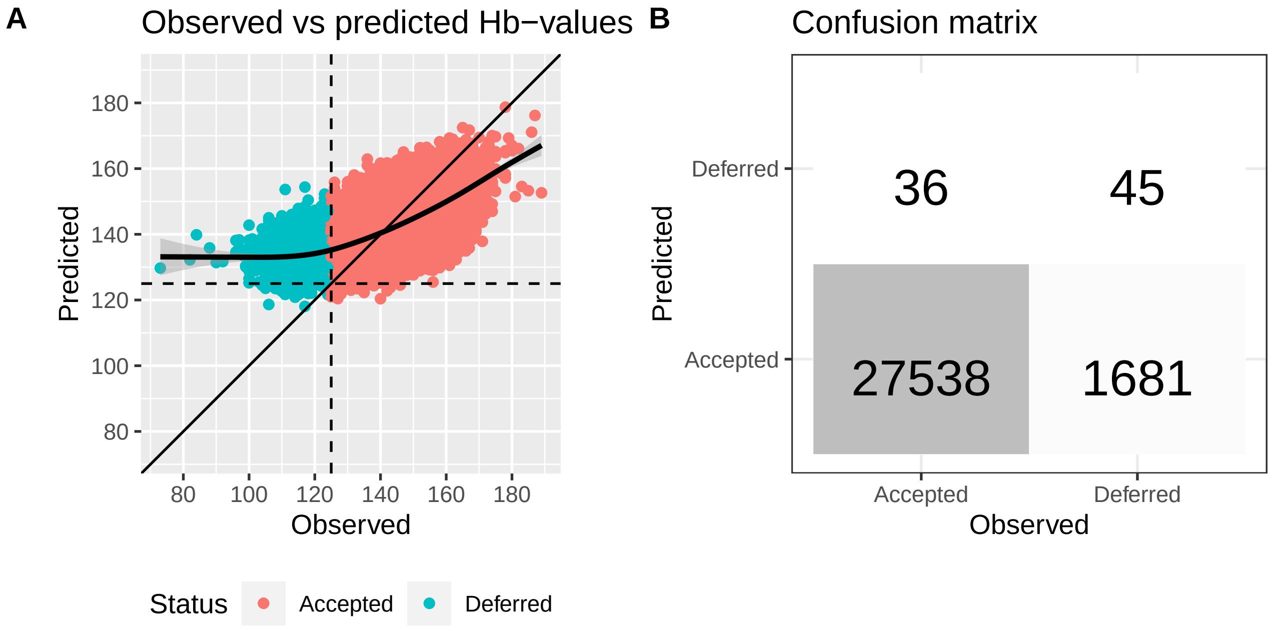

Figure S12: Classification of last donations of each female donor based on observed and predicted hemoglobin in eProgesa data. The predictions are given by the LMM. (A) The scatter plot shows the observed and predicted hemoglobin values. The dotted lines show the deferral threshold 125 g/L for females, and in solid is the $y = x$ line. (B) The confusion plot shows the classification of donations into accepted and deferred based on observed and predicted hemoglobin values.

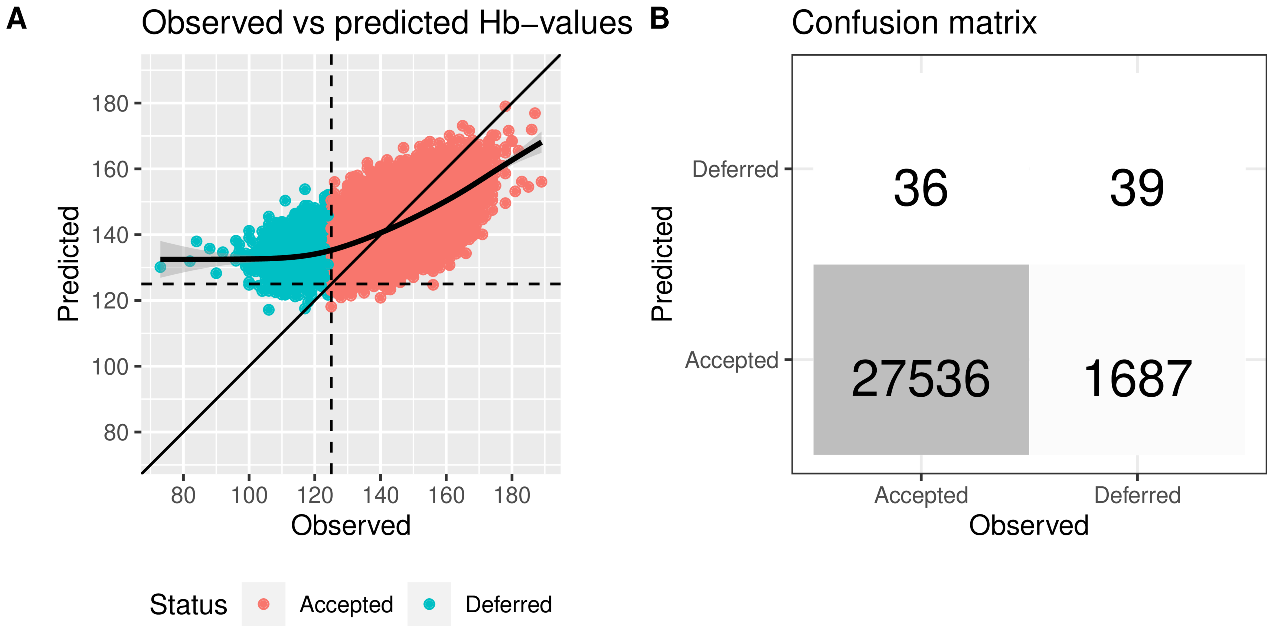

Figure S13: Classification of last donations of each female donor based on observed and predicted hemoglobin in eProgesa data using DLMM. See Figure S12 for details.

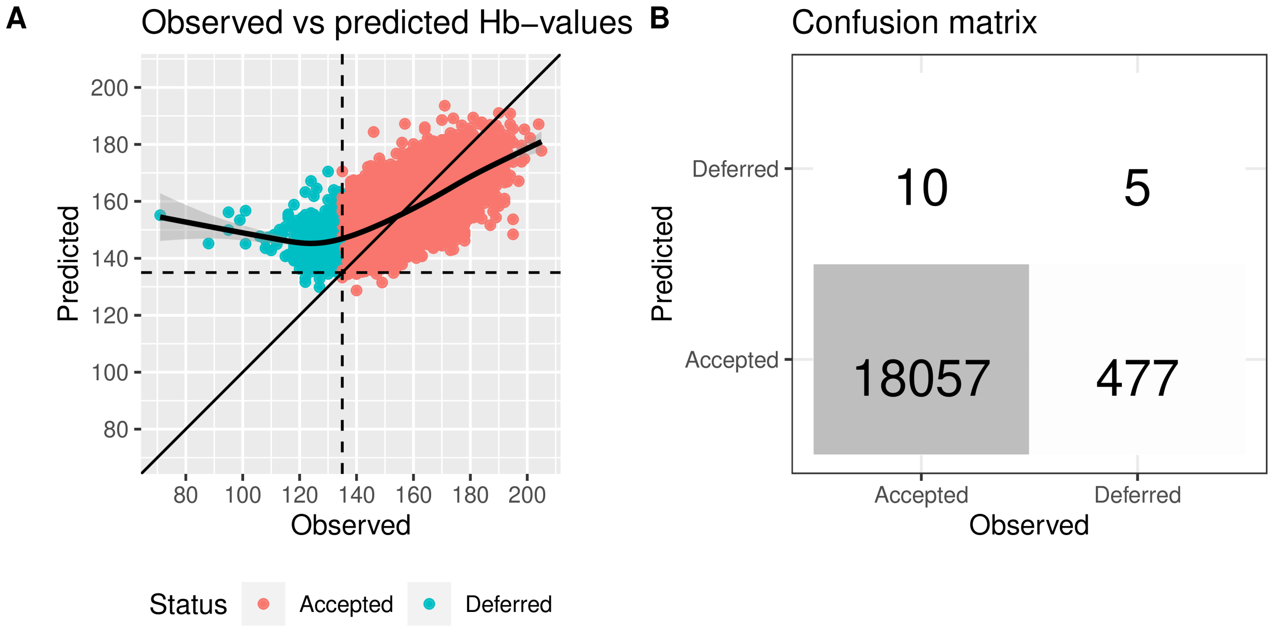

Figure S14: Classification of last donations of each male donor based on observed and predicted hemoglobin in eProgesa data using LMM. See Figure S12 for details.

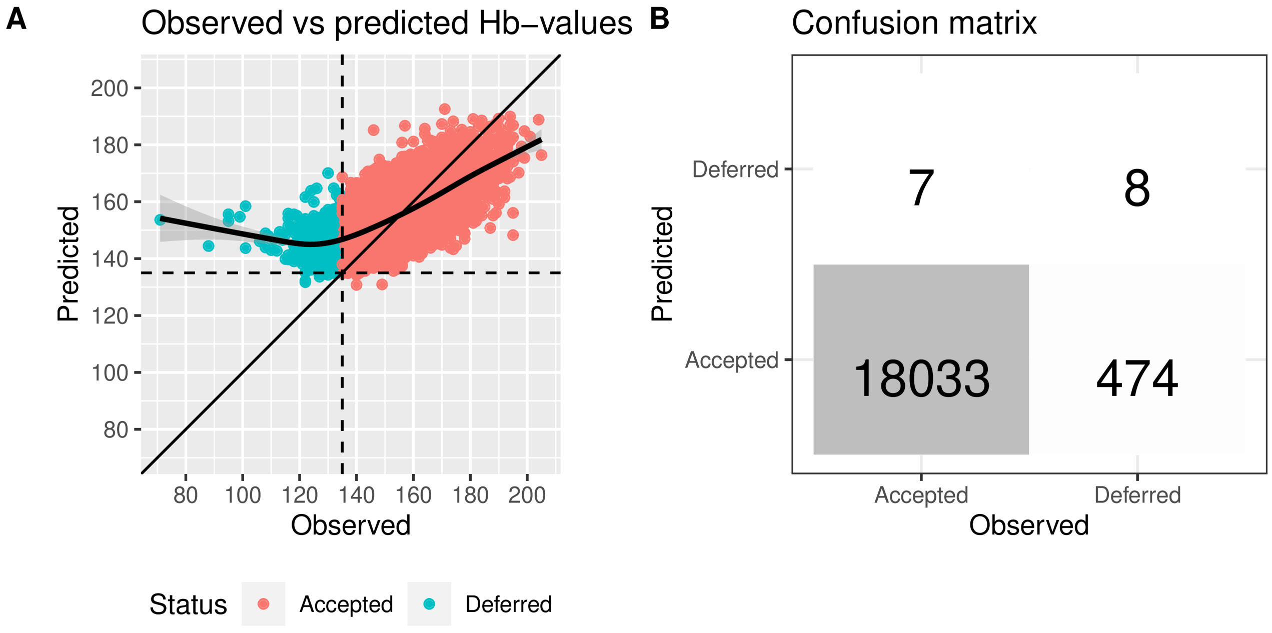

Figure S15: Classification of last donations of each male donor based on observed and predicted hemoglobin in eProgesa data using DLMM. See Figure S12 for details.

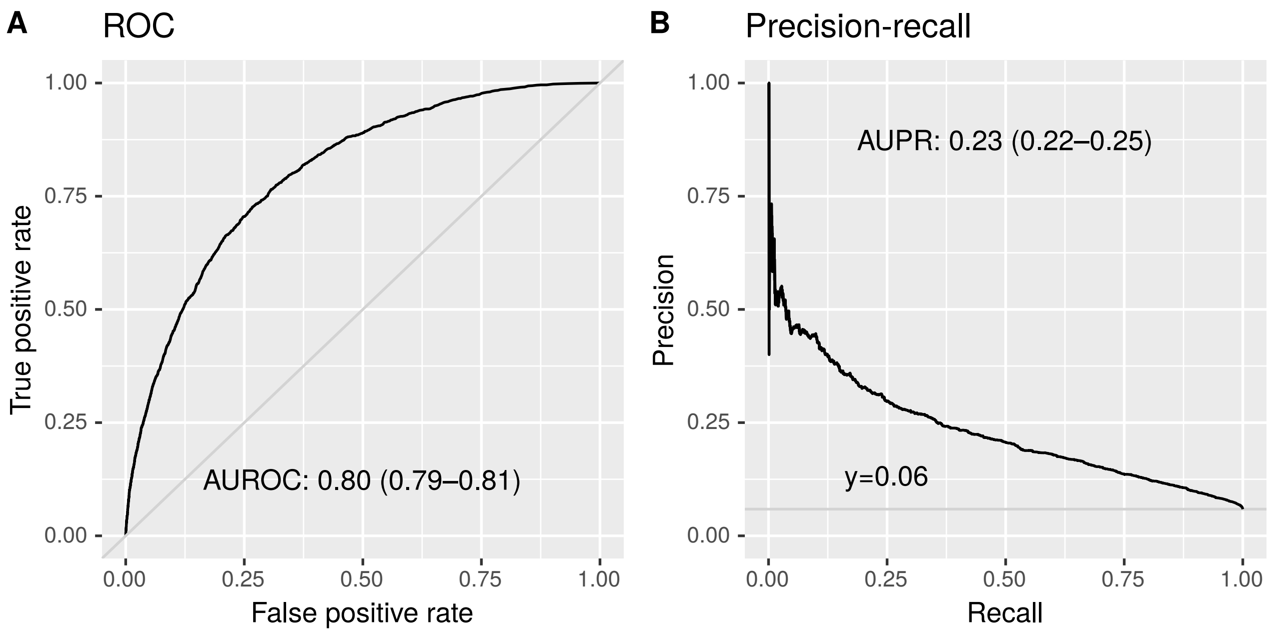

Figure S16: The classification performance of the LMM on female eProgesa data. Probability of deferral for each donor is obtained from the fitted model, and for each threshold (between 0 and 1) for the deferral probability the relevant statistics (true positive rate=recall, false positive rate, and precision) are computed, and a dot is drawn in both ROC and PR panels. (A) The receiver operating characteristic curve (ROC) and area under the curve (AUROC). The optimal ROC curve goes close to the upper left corner of the plot. (B) The precision-recall curve and the area under the curve (AUPR). The optimal PR curve goes close to the upper right corner of the plot. The higher the area under the curves, the better the classifier is. In both plots the 95% bootstrapped confidence interval of the area under the curve is shown in parentheses, and the performance curve of a random classifier is shown in gray.

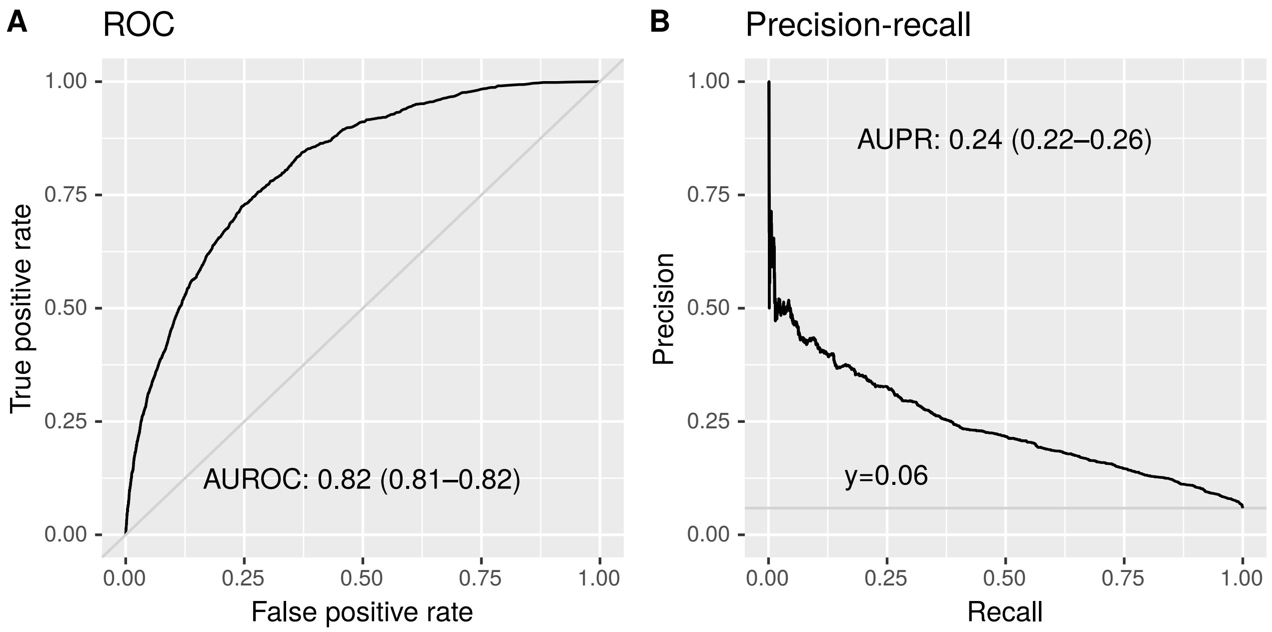

Figure S17: The classification performance of the DLMM on female eProgesa data. (A) The receiver operating characteristic curve (ROC) and area under the curve (AUROC). (B) The precision-recall curve and the area under the curve (AUPR). For details, see Figure S16.

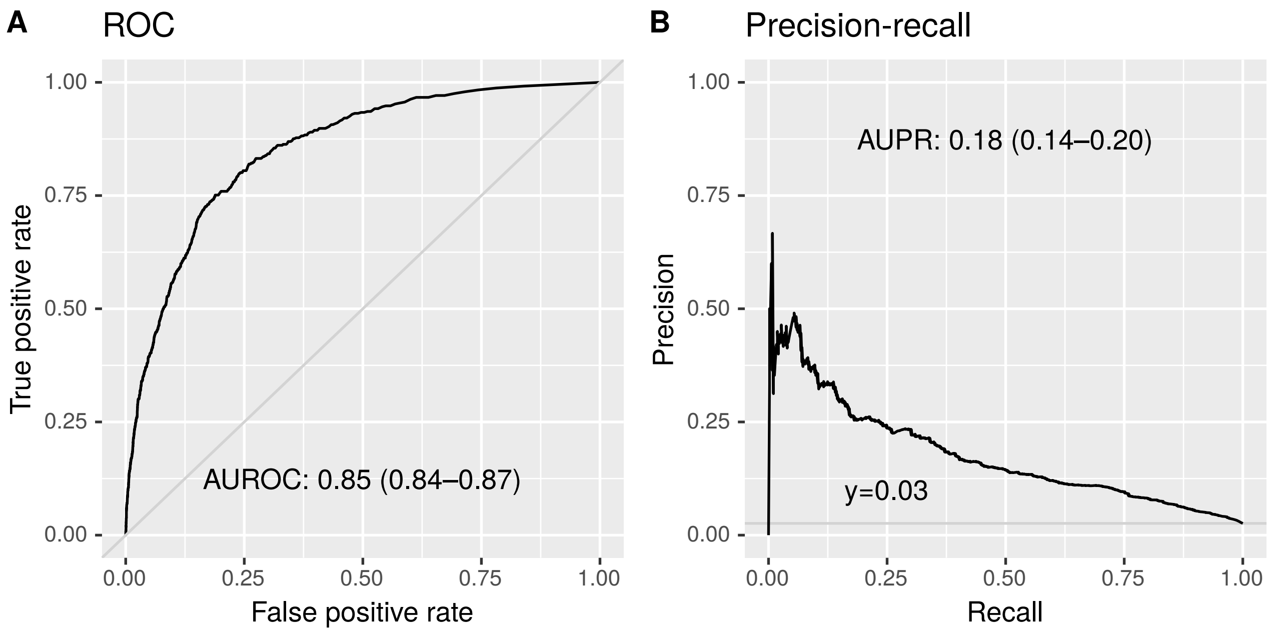

Figure S18: The classification performance of the LMM on male eProgesa data. (A) The receiver operating characteristic curve (ROC) and area under the curve (AUROC). (B) The precision-recall curve and the area under the curve (AUPR). For details, see Figure S16.

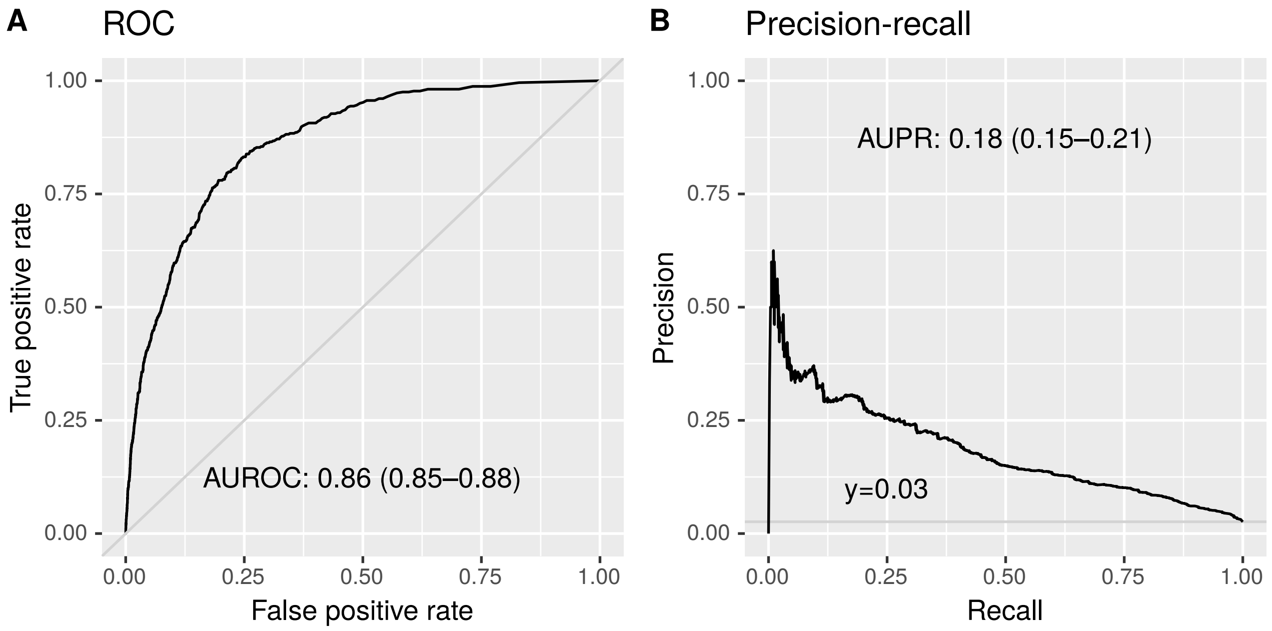

Figure S19: The classification performance of the DLMM on male eProgesa data. (A) The receiver operating characteristic curve (ROC) and area under the curve (AUROC). (B) The precision-recall curve and the area under the curve (AUPR). For details, see Figure S16.

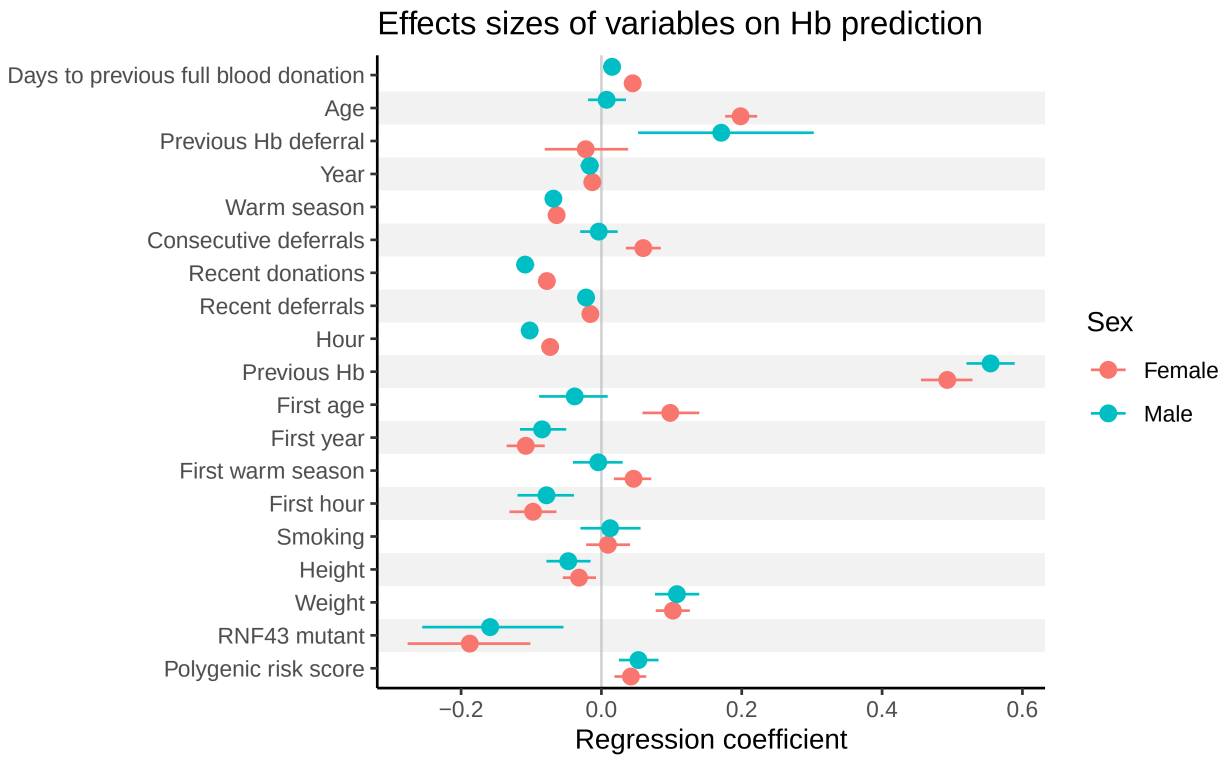

Figure S20: The effect sizes of the variables used in predicting hemoglobin of females with a DLMM on the combination of eProgesa and Biobank data sets. In addition to the mean of the posterior distribution the 95% highest posterior density interval (HPDI) is shown for each variable. See Figure S10 for details.

S3.2.3 Linear mixed models on Biobank data

Next we combined the eProgesa data with the Biobank data, which contains five donor specific variables (Table S2). Even though all the donors with Biobank information are blood donors, and hence they also have donation events in the raw eProgesa data (see Figure 1 in the main text), the dropping of donors in the proprocessing of eProgesa data means that we have to the drop the same donors also from the Biobank data. So, after merging the preprocessed eProgesa data and Biobank data, we have 12 112 donors remaining. And taking only the donors in the test half results in 6118 donors. Furthermore, requiring at least seven donations per donor gives the final data set of 4241 donors (2571 females and 1670 males).

The DLMM performed better on the eProgesa data than the LMM. The DLMM is also theoretically more appealing as it uses the whole times series data. Hence, we only fitted the DLMM to the Biobank data. The effect sizes of the resulting model are reported in Figures S20 and Table S11. As the data size is smaller than the eProgesa data the credible intervals are now a bit wider. The effects of the new variables are quite similar for both genders, and the directions of the effects are as expected. The effect of the polygenic score for hemoglobin seems rather trivial as its absolute effect size is smaller than for example the hour of donation. In contrast, the presence of the non-reference allele in the RNF43 gene has a large negative effect. For females it seems to be almost as important as age. For men it is the third most important predictor variable. However, as it is found only in around 2% of Finnish people its population level impact is nevertheless small.

Table S11: The posterior means and the HPDIs of the effect sizes from a DLMM in numeric form, the last five variables are from the Biobank data.

	**Female**			**Male**		
	**mean**	**95% CI**		**mean**	**95% CI**	
Days to previous full blood donation	0.045	0.035	0.054	0.015	0.007	0.024
Age	0.198	0.176	0.222	0.007	-0.019	0.035
Previous Hb deferral	-0.022	-0.081	0.038	0.171	0.052	0.303
Year	-0.013	-0.024	0.000	-0.017	-0.030	-0.004
Warm season	-0.064	-0.072	-0.057	-0.068	-0.076	-0.061
Consecutive deferrals	0.060	0.035	0.085	-0.004	-0.030	0.023
Recent donations	-0.078	-0.089	-0.066	-0.109	-0.120	-0.096
Recent deferrals	-0.016	-0.026	-0.005	-0.022	-0.033	-0.012
Hour	-0.073	-0.082	-0.064	-0.102	-0.111	-0.093
Previous Hb	0.493	0.455	0.529	0.555	0.520	0.589
First age	0.098	0.059	0.140	-0.038	-0.089	0.009
First year	-0.108	-0.135	-0.081	-0.085	-0.116	-0.050
First warm season	0.046	0.018	0.071	-0.004	-0.041	0.030
First hour	-0.097	-0.131	-0.064	-0.078	-0.120	-0.039
Smoking	0.009	-0.022	0.041	0.012	-0.030	0.056
Height	-0.032	-0.055	-0.008	-0.047	-0.078	-0.015
Weight	0.102	0.077	0.126	0.108	0.076	0.139
RNF43 mutant	-0.188	-0.276	-0.101	-0.159	-0.255	-0.054
Polygenic risk score	0.042	0.019	0.064	0.053	0.025	0.081

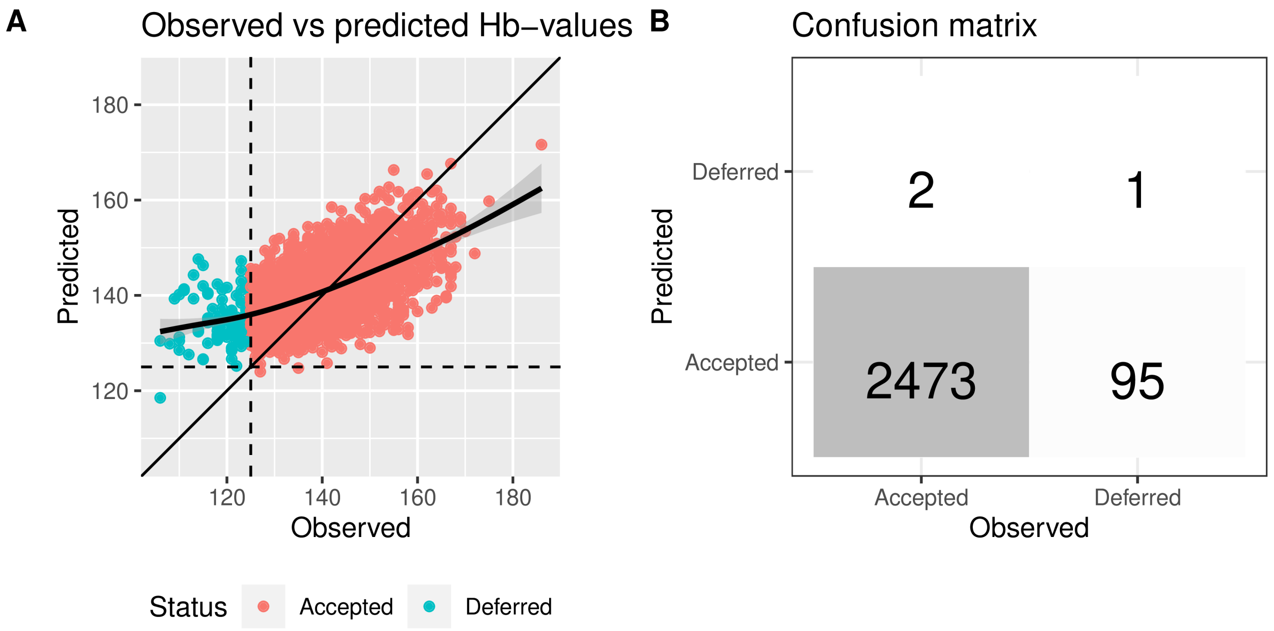

Figure S21: Classification of last donations of each female donor based on observed and predicted hemoglobin in Biobank data using DLMM. See Figure S12 for details.

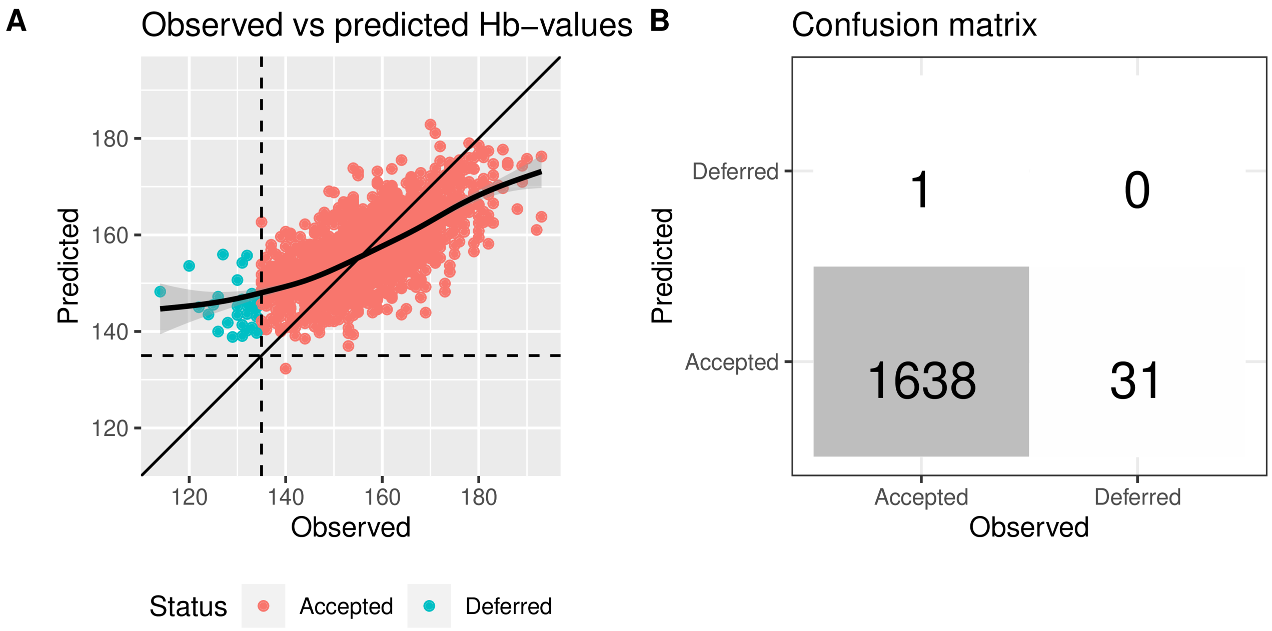

Figure S22: Classification of last donations of each male donor based on observed and predicted hemoglobin in Biobank data using DLMM. See Figure S12 for details.

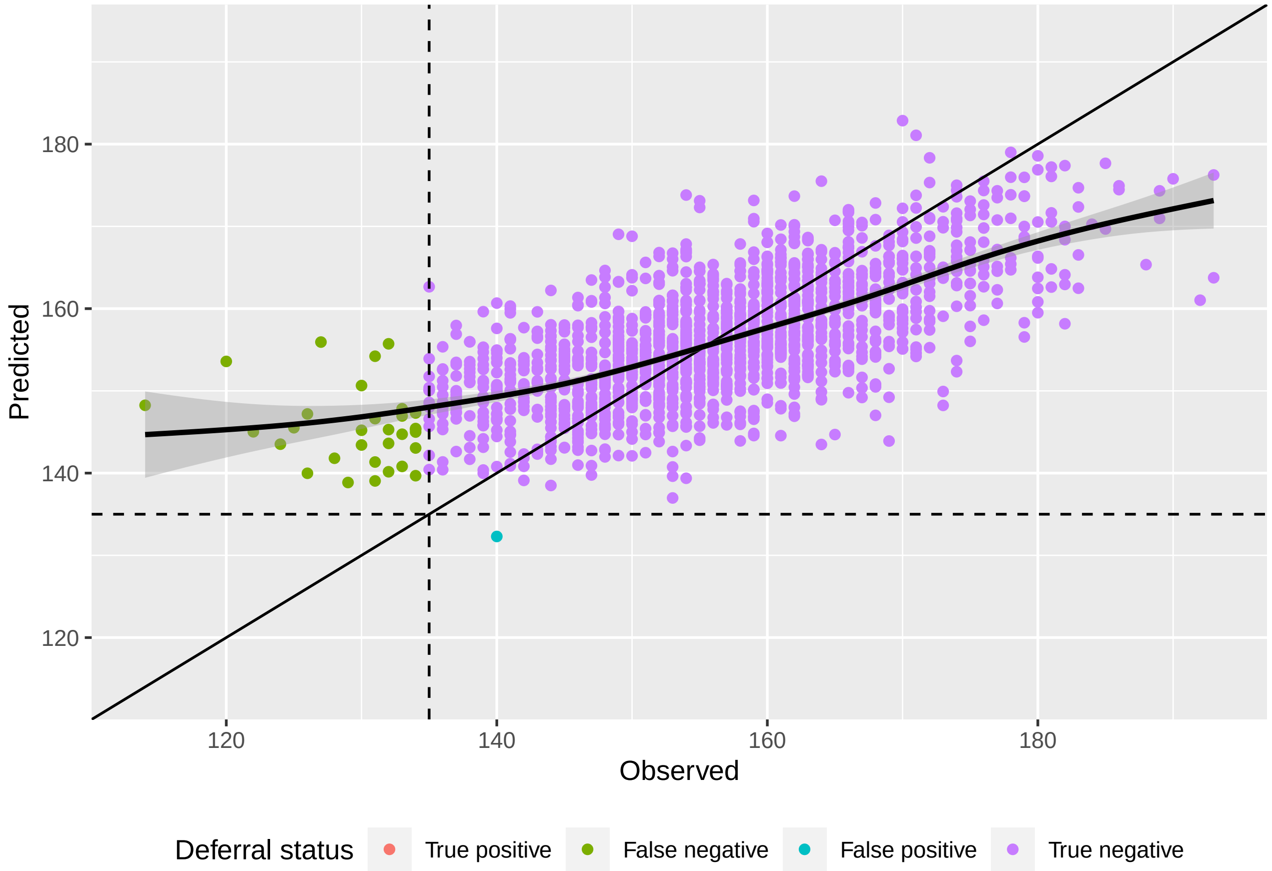

Figure S23: Classification to deferred and accepted using the posterior mean of the Biobank male DLMM and the standard male threshold 135 g/L.

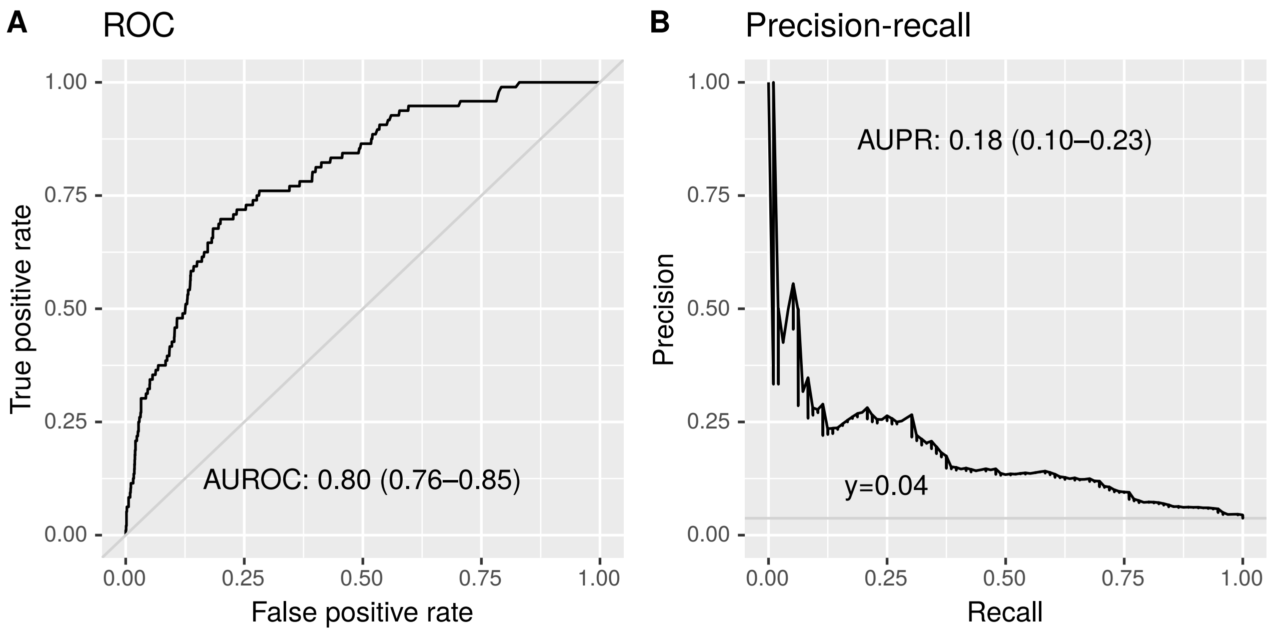

Figure S24: The classification performance of the DLMM on female Biobank data. (A) The receiver operating characteristic curve (ROC) and area under the curve (AUROC). (B) The precision-recall curve and the area under the curve (AUPR). For details, see Figure S16.

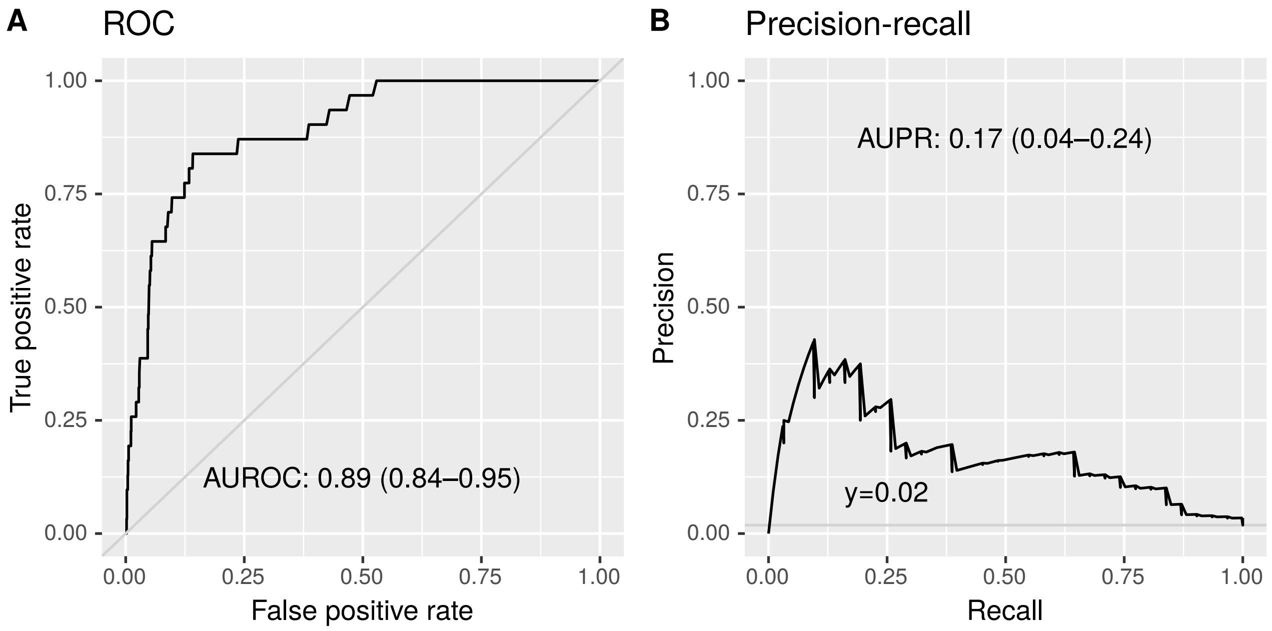

Figure S25: The classification performance of the DLMM on male Biobank data. (A) The receiver operating characteristic curve (ROC) and area under the curve (AUROC). (B) The precision-recall curve and the area under the curve (AUPR). For details, see Figure S16.

S3.2.4 Linear mixed models on FinDonor data

We selected from the FinDonor data set 11 donation specific variables describing blood levels and seven donor specific variables as new explanatory variables (see Table S3). These were merged with the full preprocessed eProgesa data. After requiring that the time series have at least length three, this resulted in a data set with 550 donors (334 female and 216 male) and 2319 donations (1293 female and 1026 male). We used full preprocessed eProgesa data, unlike with the analysis of the Biobank data, since the size of the FinDonor data set is very small. We again only fit the DLMM to the FinDonor data. When fitting the DLMM we used the 11 blood level variables from the previous event, because we cannot use the blood levels of the event we are trying to predict.

We used 4-fold cross-validation and out-of-sample prediction, that is, prediction of hemoglobin of donors whose data was not used in fitting of the model, to avoid overfitting. The small data size, especially for men, and the large number of variables make inference hard as the large credible intervals in Figures S26 and Table S12 indicate. In addition, the female model fitting did not converge even though 20 000 iterations per chain was used. To get more credible results, a subset of variables should be used or more data should be obtained.

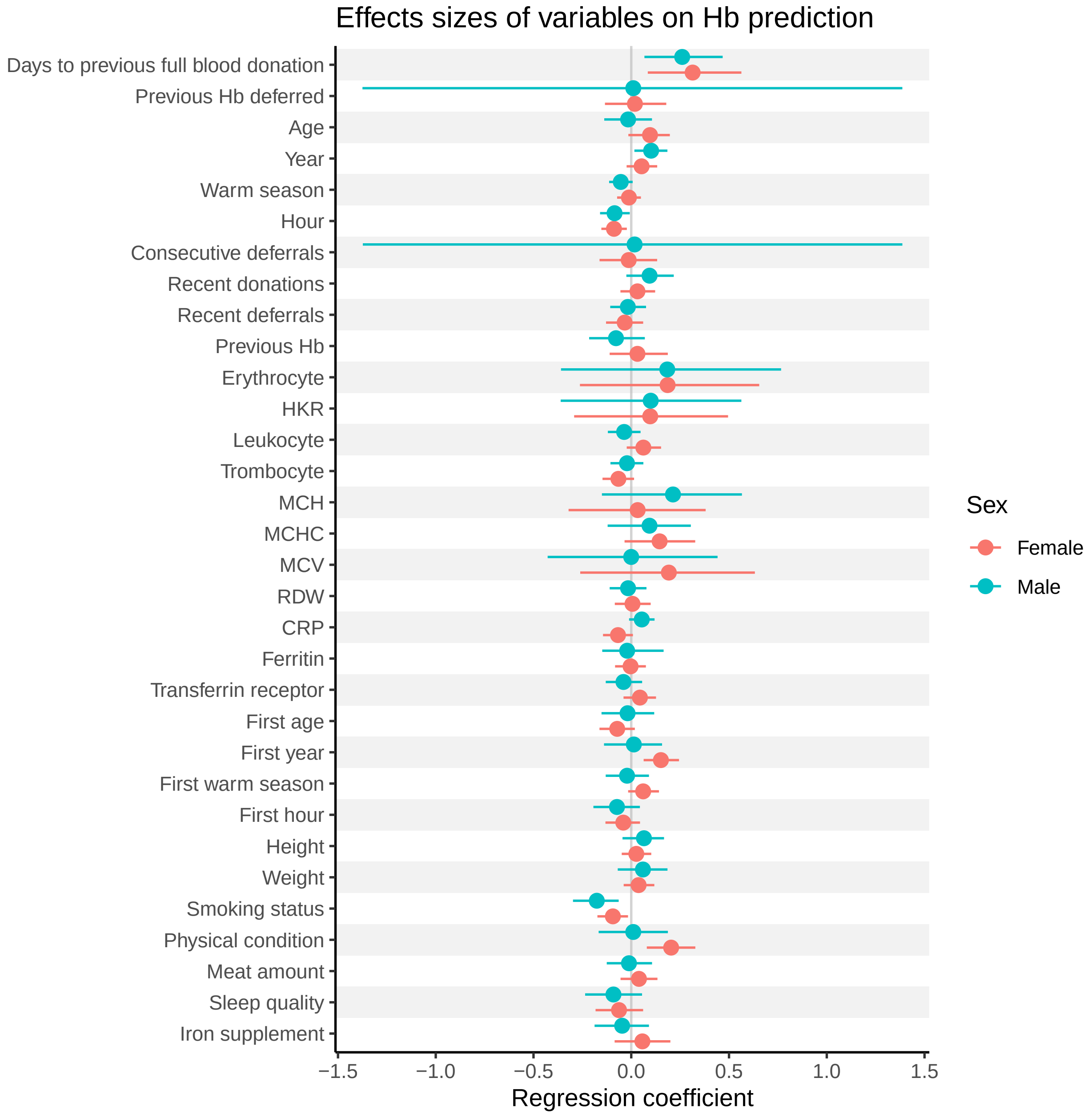

Figure S26: The effect sizes of the variables used in predicting hemoglobin with a DLMM on the combination of eProgesa and FinDonor data sets. In addition to the mean of the posterior distribution the 95% highest posterior density interval (HPDI) is shown for each variable. See Figure S10 for details.

Table S12: The effect sizes of the variables used in predicting hemoglobin with DLMMs on the combination of eProgesa and FinDonor data. The posterior means and 95% highest posterior density intervals are shown for each variable. The first ten variables are from eProgesa, the next eleven variables are the lagged blood levels from FinDonor data, the following four variables are used in modeling the connection between the donor specific intercepts and the first hemoglobin values in the ICP correction (see Equation S2), and the last seven variables are the donor specific variables from FinDonor.

	**Female**			**Male**		
	**mean**	**95% CI**		**mean**	**95% CI**	
Days to previous full blood donation	0.314	0.085	0.563	0.260	0.067	0.468
Previous Hb deferred	0.019	-0.135	0.179	0.010	-1.375	1.387
Age	0.096	-0.015	0.197	-0.016	-0.138	0.106
Year	0.053	-0.024	0.133	0.102	0.016	0.185
Warm season	-0.012	-0.072	0.049	-0.054	-0.113	0.007
Hour	-0.088	-0.153	-0.023	-0.085	-0.160	-0.008
Consecutive deferrals	-0.013	-0.162	0.132	0.017	-1.373	1.387
Recent donations	0.032	-0.055	0.122	0.094	-0.025	0.217
Recent deferrals	-0.034	-0.129	0.061	-0.017	-0.108	0.076
Previous Hb	0.032	-0.111	0.187	-0.078	-0.215	0.069
Erythrocyte	0.186	-0.263	0.654	0.184	-0.359	0.766
HKR	0.097	-0.292	0.495	0.099	-0.361	0.563
Leukocyte	0.062	-0.023	0.152	-0.036	-0.120	0.047
Thrombocyte	-0.066	-0.147	0.014	-0.022	-0.106	0.062
MCH	0.033	-0.321	0.380	0.213	-0.150	0.566
MCHC	0.145	-0.034	0.327	0.093	-0.121	0.305
MCV	0.192	-0.261	0.632	0.000	-0.428	0.441
RDW	0.006	-0.084	0.099	-0.016	-0.110	0.078
CRP	-0.068	-0.145	0.009	0.054	-0.011	0.119
Ferritin	-0.004	-0.083	0.075	-0.021	-0.148	0.165
Transferrin receptor	0.044	-0.040	0.127	-0.040	-0.131	0.056
First age	-0.072	-0.163	0.018	-0.019	-0.152	0.117
First year	0.152	0.063	0.244	0.013	-0.139	0.158
First warm season	0.061	-0.016	0.142	-0.022	-0.131	0.090
First hour	-0.041	-0.132	0.045	-0.073	-0.194	0.044
Height	0.026	-0.049	0.102	0.065	-0.045	0.168
Weight	0.038	-0.039	0.118	0.060	-0.069	0.185
Smoking status	-0.094	-0.173	-0.017	-0.176	-0.298	-0.064
Physical condition	0.204	0.079	0.328	0.010	-0.167	0.187
Meat amount	0.039	-0.055	0.134	-0.012	-0.125	0.106
Sleep quality	-0.063	-0.183	0.061	-0.091	-0.236	0.055
Iron supplement	0.057	-0.085	0.200	-0.047	-0.188	0.091

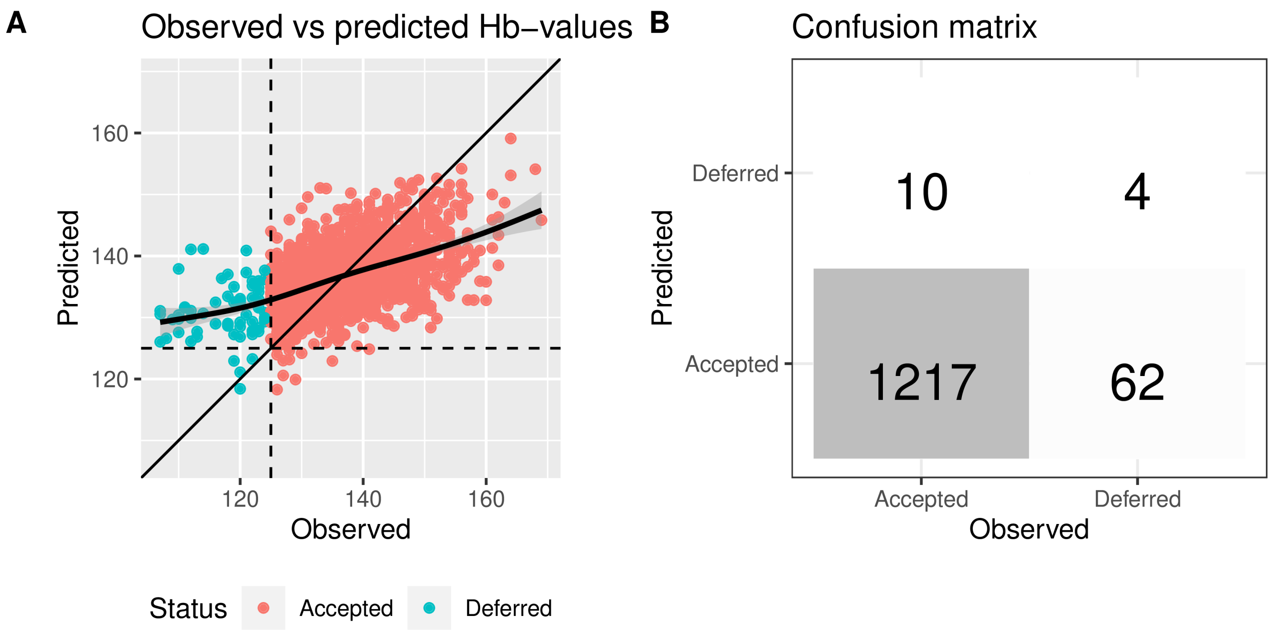

Figure S27: Classification of last donations of each female donor based on observed and predicted hemoglobin in FinDonor data using DLMM. See Figure S12 for details.

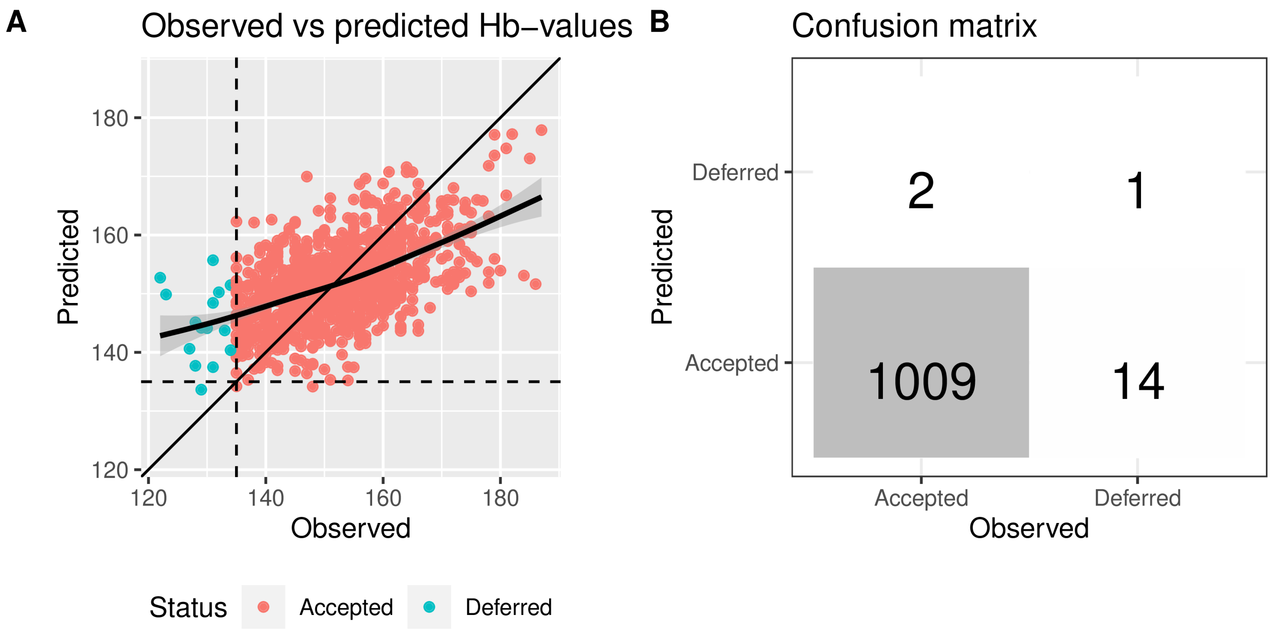

Figure S28: Classification of last donations of each male donor based on observed and predicted hemoglobin in FinDonor data using DLMM. See Figure S12 for details.

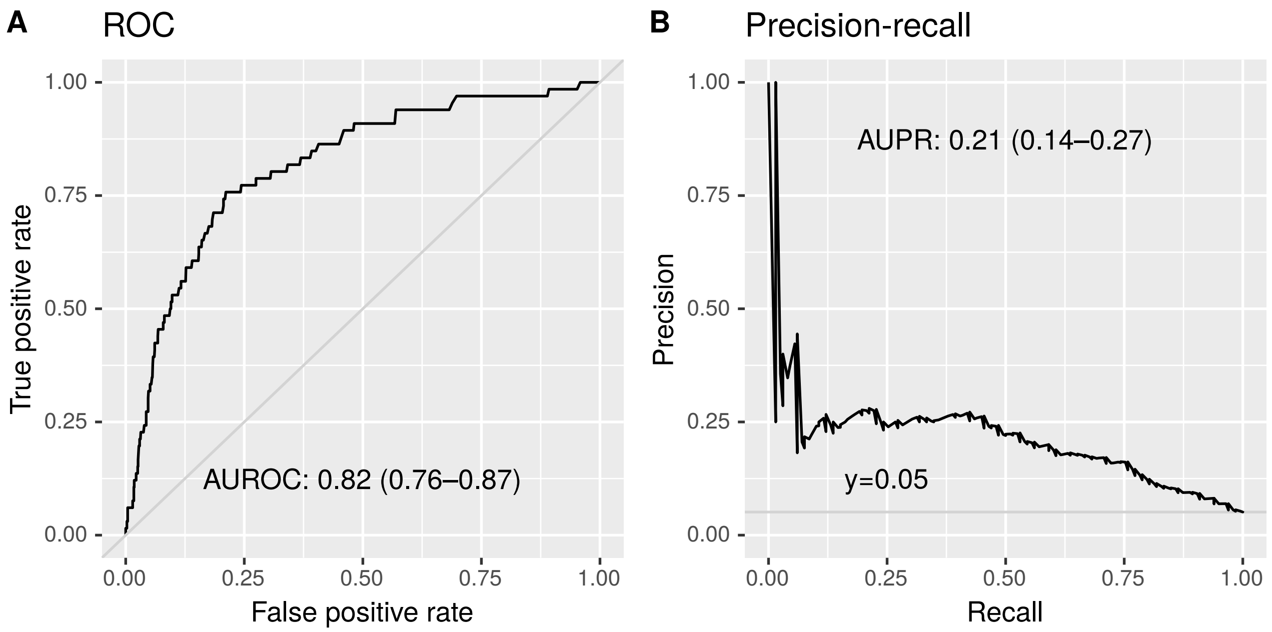

Figure S29: The classification performance of the DLMM on female FinDonor data. (A) The receiver operating characteristic curve (ROC) and area under the curve (AUROC). (B) The precision-recall curve and the area under the curve (AUPR). For details, see Figure S16.

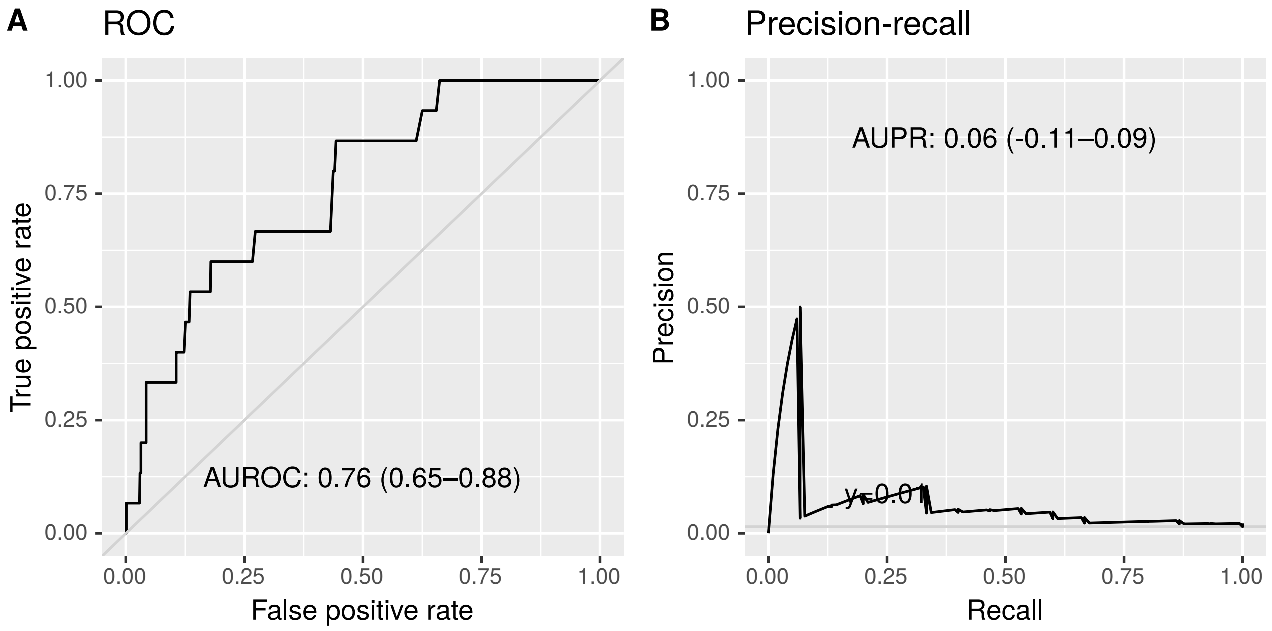

Figure S30: The classification performance of the DLMM on male FinDonor data. (A) The receiver operating characteristic curve (ROC) and area under the curve (AUROC). (B) The precision-recall curve and the area under the curve (AUPR). For details, see Figure S16.

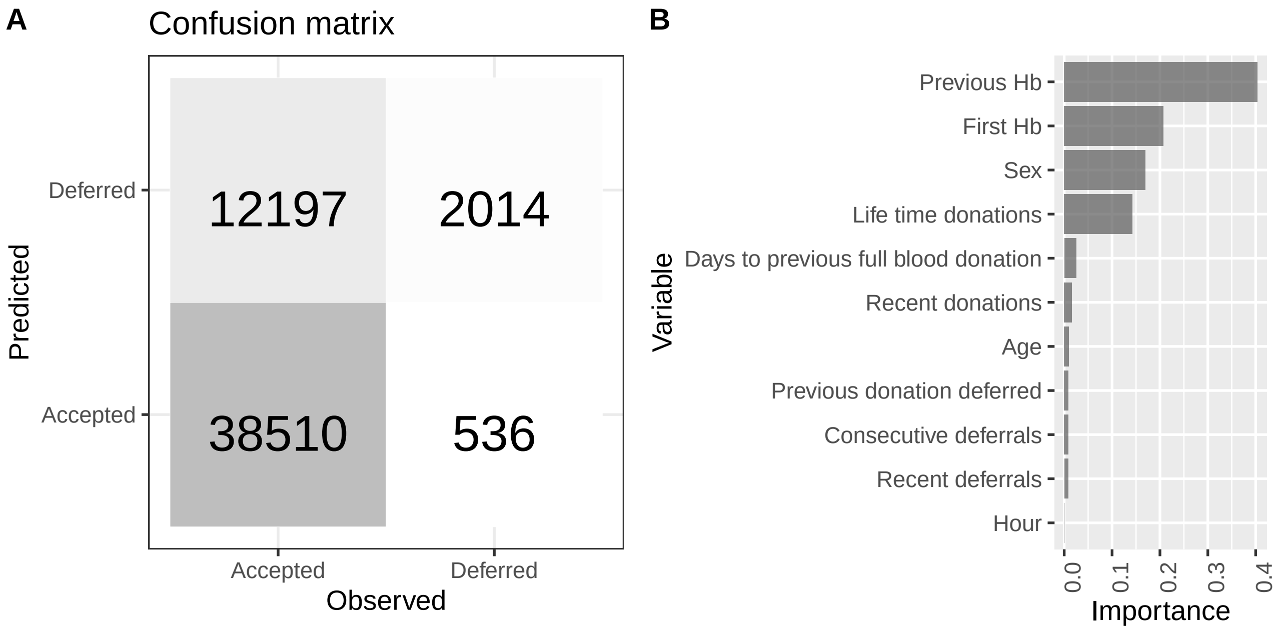

Figure S31: Confusion matrix (A) and variable importances (B) of the decision tree model.

S3.2.5 Random forest on eProgesa data

To find a baseline level for the random forest model we first fit a single decision tree using only eProgesa data. The complexity parameter `cp` was found to be 0.00453 (using search grid of ten equally spaced values in the range $[0.0001, 0.04]$). The confusion matrix shows a true positive rate, i.e. sensitivity, of over 79% (Figure S31 A). In comparison, in the LMM model's the true positive rate is consistently below 10%. As in the DLMMs for eProgesa (donation histories) and Biobank (smoking status, weight, height and genotype) data, the previous hemoglobin measurement is the most important predictor of deferral (Figure S31 B). For the LMMs we use model coefficients as a measure of importance of variable. For binary classifiers we use 'variable importance' that measures how much a model uses a variable to make accurate predictions. Hence, they can be compared only by how they rank the variables. The second most important is the first hemoglobin measurement which is the most important predictor in the LMM for eProgesa data. Sex is third most important predictor of deferral. Accordingly, we tested also fitting a separate model for men and women but found no noticeable performance gains. Figure S32 shows the ROC and precision-recall curves of the decision tree. It is of note that both show lower AUC values than all of the LMMs except for the FinDonor model on male data (Figure S30). Figure S33 shows the final decision tree selected by the algorithm. It uses only four variables "Previous Hb", "Number of lifetime donations", "First Hb", and "Recent donations". In the Figure S31 B also other variables are shown to be important. This is because the variable importance calculation includes information from variables that were tested for splitting a node, but were not finally used. Because of correlations between variables, other than the variables in the final tree, might as well have been used [Ste09]. Indeed, when developing the code, we found that minor changes in the data result in slightly different decision trees.

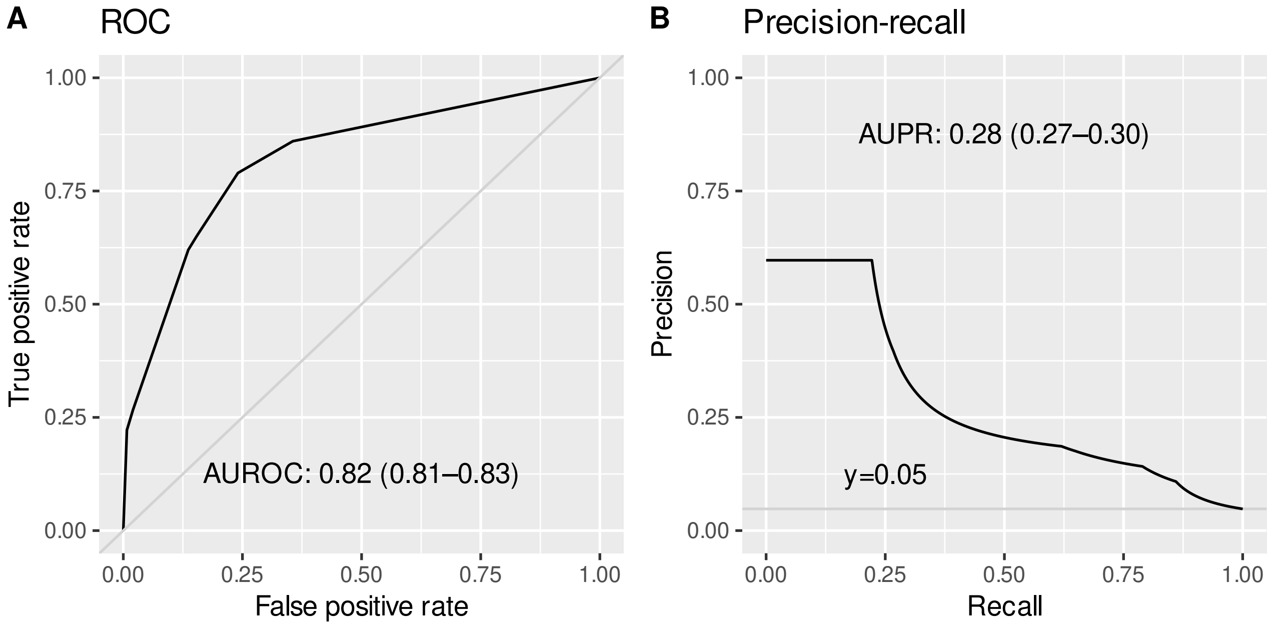

Figure S32: Classification performance of decision tree. See Figure S16 for further details.

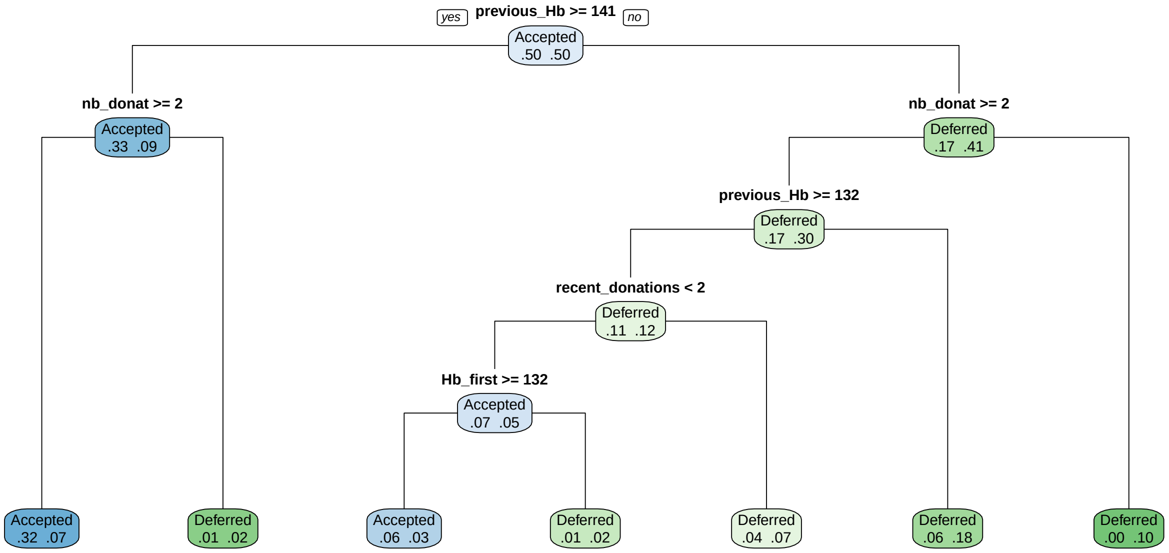

Figure S33: Decision tree on eProgesa data. The tree is read starting from the top. Is the previous hemoglobin of a donor at least 141 and number of lifetime donations at least two? If yes, the tree predicts that he or she should be admitted to donate. The proportions at nodes are proportions of all donors i.e. 7% of donors are accepted to donate based on having previous hemoglobin of at least 142 and number of lifetime donations at least two, but are in reality deferred, while 32% were in reality accepted.

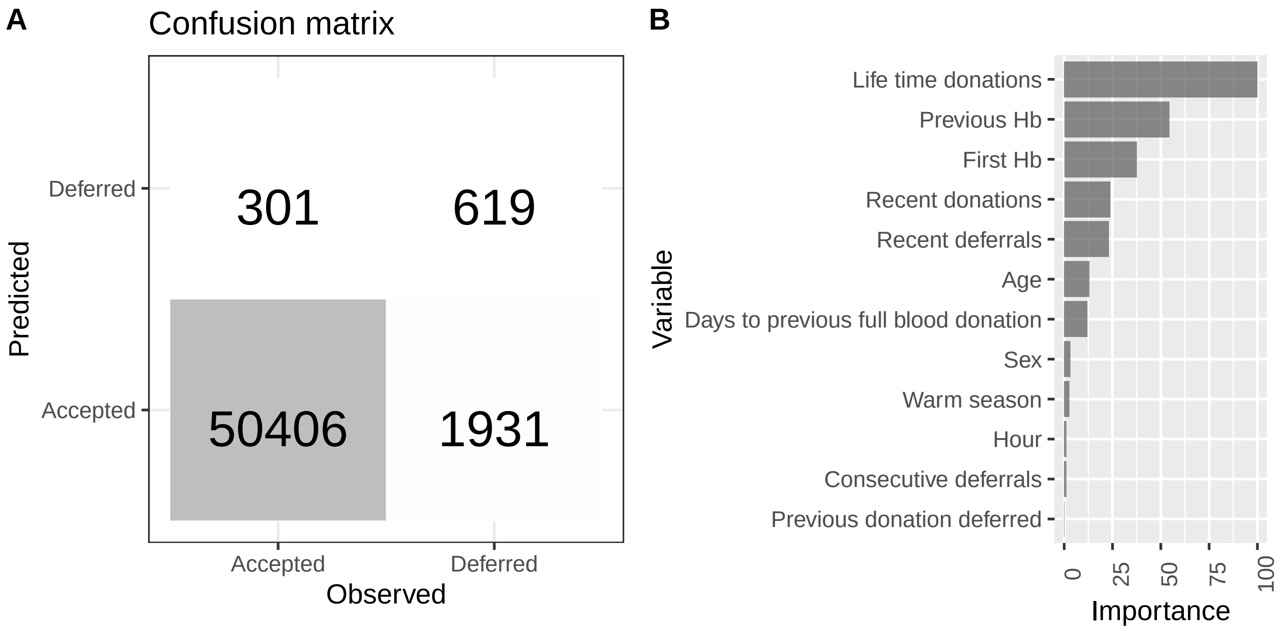

Figure S34: Confusion matrix (A) and variable importances (B) of the random forest model.

To fit the random forest model, we first tuned the random forest hyperparameter by a grid search. The value 4 from range 3–8 for hyperparameter `mtry` (number of random variables tested for splitting a node) was found to give best performance in the cross-validation process with the training data.

We then validated the model using validation data that was not used in the training process. The confusion matrix again, as in the decision tree, shows a true positive rate of 24% (Figure S34 A). The variable importance of "Lifetime donations" is the highest, while the variable "Previous Hb" is the second highest (Figure S34 B). Sex does not appear as an important predictor. Nevertheless, we tested also separate models for the two sexes, but found no noticeable improvement. The AUROC and AUPR were found to be higher than for the decision tree (Figure S35).

Below, in addition to the true positive rate, we use the false positive rate for estimation of economic effects of using a prediction model in blood banking. The confusion matrix of the random forest shows a false positive rate of over 0.58% (Figure S34). To try to understand where does this rate stem from we inspected density distributions of all the variables divided by the quadrants of the confusion matrix (Figure S36). In the variable a "Previous Hb" a clear split is seen. Donors that were accepted to donate and were predicted to be accepted for donation (true negative) have the highest mean hemoglobin. The donors that were deferred, but predicted to be accepted (false negative) have also a higher previous hemoglobin compared to the donors that were deferred and were predicted to be deferred (true positive). A similar difference is seen concerning correct acceptance prediction, but vice versa in regard to hemoglobin values.

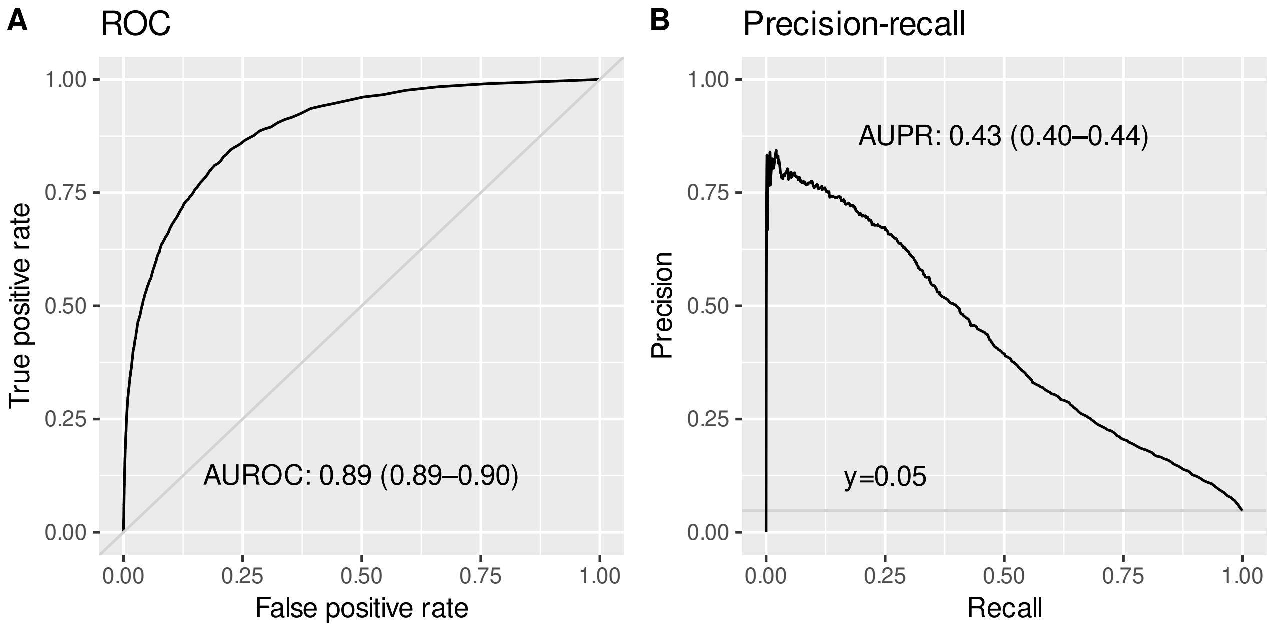

Figure S35: Classification performance of random forest. See Figure S16 for further details.

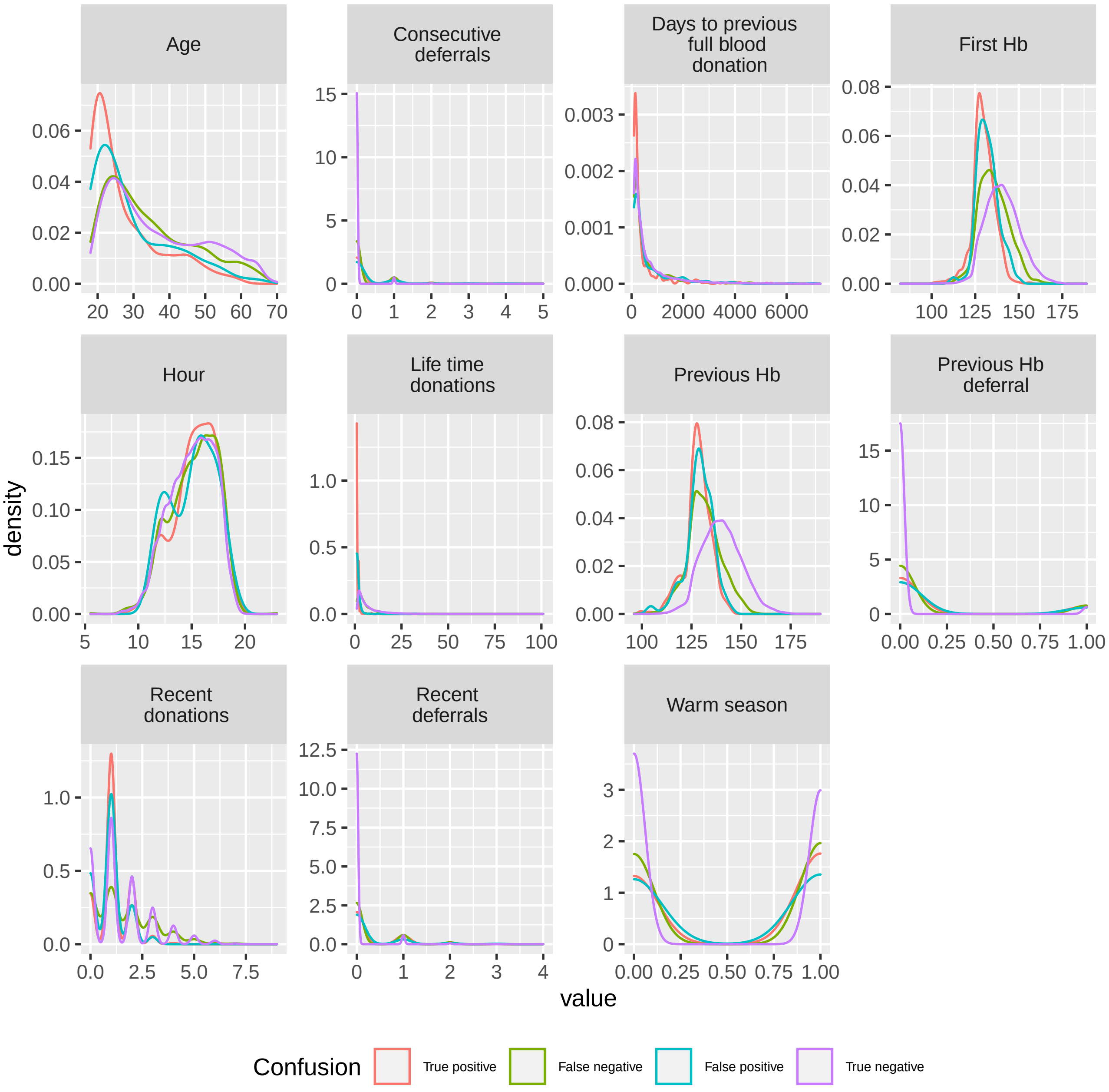

Figure S36: Density distributions of all predictor variables for women in the validation data set used in random forest divided by confusion matrix quadrants. Similar results were seen also for men.

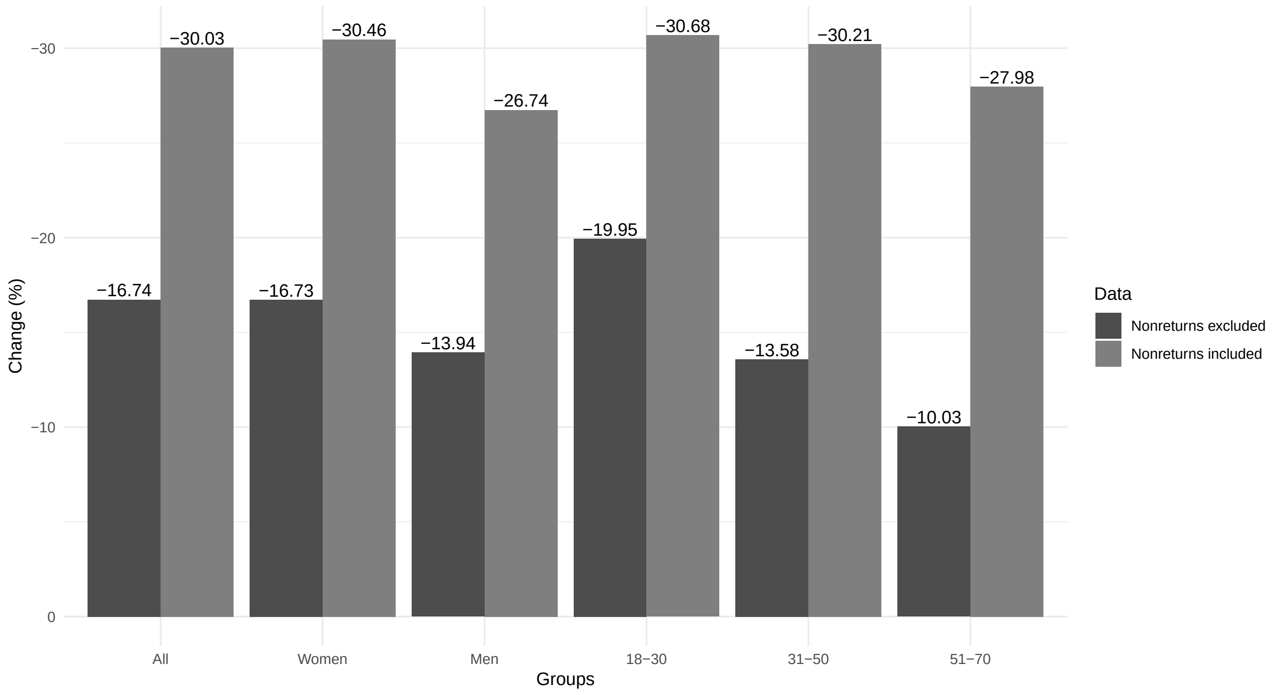

Figure S37: Effect of an Hb deferral on donation frequency. Hb deferral decreases donor frequency by 16.7% on average when frequency is measured before and after the first Hb deferral. The effect size almost doubles if we include donors who do not return after their first deferral.

S3.3 Estimation of financial and blood supply effects

S3.3.1 Estimation of the effect of Hb deferral on donor retention

The potential negative effects of Hb deferrals on donor retention were estimated by examining donation frequencies (visits/year) among donors, before and after receiving a deferral due to low Hb. The last 10 years of the eProgesa data was used for this analysis. Several groups of donors were removed from the data for this analysis, and we included only donors that had not had a Hb deferral before our 10 year target window, had at least one Hb deferral but not any other kinds of deferrals, had donated at least 3 times, and whose first deferral was not their first event in our target window. We calculated two different frequencies: one with only the donors that returned at least once after receiving a deferral, and a second one with non-returning donors included. Donation frequencies were calculated first for all and then separately for women, men, and different age groups. The mean change in frequency after first Hb deferral received by donor is presented in Figure S37. The total effect of Hb deferral on donor retention during the past 10 years is around -17% (from 2.456 events/year to 2.045 events/year) when we examine only donors that did return to donate. The effect grows to -30% if we include non-returning donors as zeroes in the mean frequency calculation. These results are corroborated by earlier published literature [HBN98; Cus+07; Hil+11; SRS21].

S3.3.2 Cost effect at the FRCBS

Significant part of the surface is negative, indicating that savings are easily gained when the total adjustment coefficient stays relatively small. All cost effects relating to model performance can be found following the plotted surface. For example, if the model extends the average donation interval by **1.1** (about 100 days instead of 91 days for women and 67 instead of 61 for men) and it would reduce the deferral rate from **3.2 percent to 2 percent** ($q = 1.2/3.2 = 0.375$), it would have a negative (saving) cost effect of **-0.05 €** per collected whole blood unit. The cost surface minimum, at right hand lower corner of the plot i.e. all deferrals avoided with no extension of donation intervals, is -0.68 €.

The random forest model outputs probabilities of deferrals. To produce Figure S34, and for all other method development, a standard cut-off of 50% was used i.e. if the model predicted higher than 50% probability of deferral then the donor was classified to be deferred. To test the effect of applying other cut-offs we classified

the predictions of the random forest model with varying probabilities and carried out the cost effect calculation for each value (Figure S39). We find that for the 6 month deferral scenario a cut-off of 20% and the 12 month scenario 40% cut-off produces largest savings. The amount in euros per donation is shown as white dots on the cost surface (Figure S38).

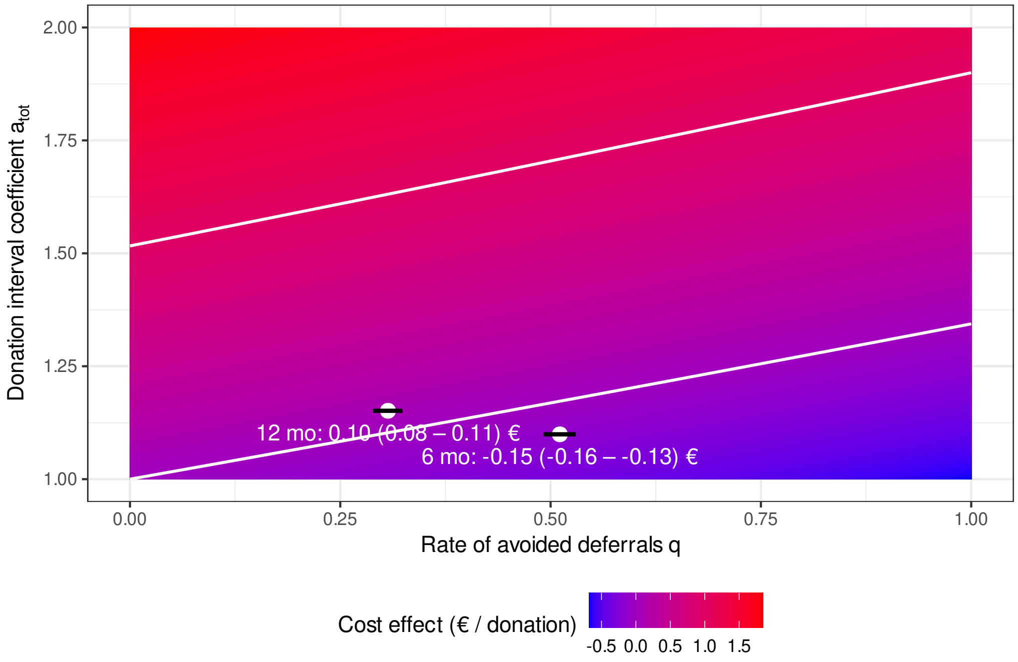

Figure S38: Cost surface drawn using the equation for E_M in the main text and values given in Table 1. Values are extracted from historical data between 2018 and 2020. q is the rate of avoided deferrals and adjustment a_{tot} is the total (average) adjustment to the donation intervals in the donor population. The white dots signify the cost effect of our classifier when deferrals extend the donation interval either to 6 or 12 months. 95% confidence interval of q, a_{tot} and the cost effect, calculated by bootstrapping model predictions (as for AUROC and AUPR values), are shown. They try to capture the variation caused by applying the model to different validation data sets. White lines signify $-1, 0$ and 1€ cost effects.

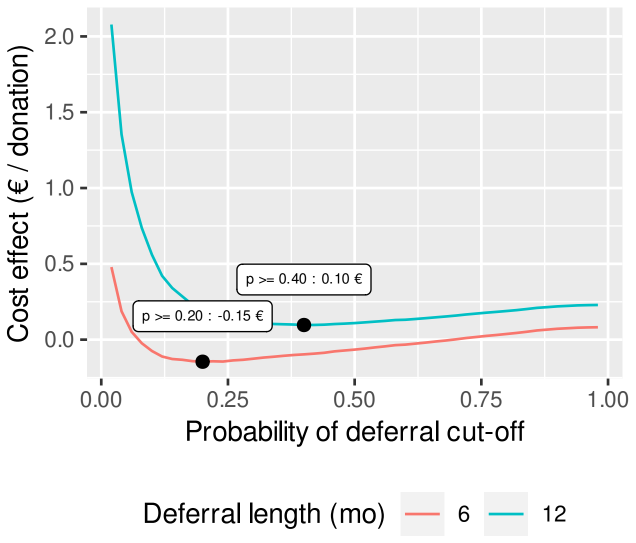

Figure S39: On y-axis cost effect of the random forest model. On x-axis probability cut-off used to define a deferral from predictions of the random forest model. Different lines show the 6 month and 12 month deferral scenarios.

Table S13: Hemoglobin prediction errors for each data set and model. To ease comparison to previous literature the mean absolute error (MAE) and root mean square error (RMSE) are given in two commonly used hemoglobin measurement units.

			g/L		mmol/L	
**Data**	**Gender**	**Model**	**MAE**	**RMSE**	**MAE**	**RMSE**
eProgesa	Male	LMM	6.67	8.60	0.41	0.53
eProgesa	Female	LMM	6.73	8.64	0.42	0.54
eProgesa	Male	DLMM	6.56	8.46	0.41	0.53
eProgesa	Female	DLMM	6.67	8.54	0.41	0.53
FinnGen	Male	DLMM	6.42	8.20	0.40	0.51
FinnGen	Female	DLMM	6.58	8.33	0.41	0.52
FinDonor	Male	DLMM	6.94	8.74	0.43	0.54
FinDonor	Female	DLMM	6.33	8.02	0.39	0.50
eProgesa	Both	Decision tree	-	-	-	-
eProgesa	Both	Random forest	-	-	-	-

Table S14: The numeric values of the classification performance metrics and their 95% confidence intervals in Fig. 3.

	AUROC			AUPR			F1			Threshold		E6			E12		
Id	value	low	high	value	low	high	value	low	high	6	12	value	low	high	value	low	high
progesa-male-lmm	0.85	0.84	0.87	0.18	0.14	0.21	0.02	0.00	0.03	0.24	0.34	0.02	0.02	0.03	0.08	0.07	0.08
progesa-female-lmm	0.80	0.79	0.81	0.23	0.21	0.25	0.04	0.03	0.05	0.14	0.32	-0.09	-0.10	-0.07	0.12	0.11	0.13
progesa-male-dlmm	0.86	0.85	0.88	0.18	0.15	0.21	0.01	0.00	0.02	0.20	0.42	0.02	0.02	0.03	0.08	0.07	0.08
progesa-female-dlmm	0.82	0.81	0.82	0.24	0.22	0.26	0.03	0.02	0.04	0.12	0.28	-0.10	-0.11	-0.09	0.12	0.11	0.13
finngen-male-dlmm	0.89	0.84	0.95	0.17	0.05	0.27	NA	NA	NA	0.26	0.26	0.01	-0.01	0.04	0.08	0.05	0.10
finngen-female-dlmm	0.80	0.76	0.85	0.18	0.11	0.24	0.04	-0.01	0.08	0.12	0.24	-0.11	-0.16	-0.05	0.10	0.04	0.15
findonor-male-dlmm	0.76	0.64	0.88	0.06	-0.07	0.15	0.11	-0.09	0.19	0.54	0.54	0.02	0.00	0.04	0.07	0.05	0.09
findonor-female-dlmm	0.82	0.77	0.87	0.21	0.13	0.29	0.10	0.01	0.19	0.20	0.30	-0.12	-0.19	-0.06	0.13	0.06	0.20
progesa-both-dt	0.82	0.81	0.83	0.28	0.27	0.30	0.24	0.23	0.25	0.80	0.80	-0.05	-0.06	-0.04	0.14	0.12	0.15
progesa-both-rf	0.89	0.89	0.90	0.43	0.40	0.45	0.36	0.34	0.38	0.20	0.40	-0.15	-0.16	-0.13	0.10	0.08	0.11
progesa-male-baseline	0.83	0.82	0.85	0.11	0.09	0.13	0.16	0.14	0.17	0.88	0.96	0.03	0.02	0.03	0.08	0.08	0.08
progesa-female-baseline	0.77	0.76	0.78	0.18	0.17	0.20	0.20	0.19	0.20	0.76	0.96	-0.05	-0.06	-0.04	0.15	0.14	0.15

References

[Bäc+20] S. Bäckman et al. "Venous sample is superior to repeated skin-prick testing in blood donor haemoglobin second-line screening". In: *Vox Sang.* (2020).

[Bre01] Leo Breiman. "Random Forests". In: *Machine Learning* 45.1 (2001), pp. 5–32.

[Car+17] Bob Carpenter et al. "Stan: A Probabilistic Programming Language". In: *Journal of Statistical Software, Articles* 76.1 (2017), pp. 1–32. DOI: 10.18637/jss.v076.i01.

[Cas+18] Johanna Castrén et al. "The impact of analytical variation of hemoglobin measurement on blood donors' hemoglobin and deferral rates". In: *Transfusion* 58.9 (2018), pp. 2157–2165. DOI: https://doi.org/10.1111/trf.14825.

[Cha+15] Christopher C Chang et al. "Second-generation PLINK: rising to the challenge of larger and richer datasets". In: *Gigascience* 4.1 (2015), s13742–015.

[CR20] Angelo Canty and B. D. Ripley. *boot: Bootstrap R (S-Plus) Functions*. R package version 1.3-25. 2020.

[Cus+07] Brian Custer et al. "The consequences of temporary deferral on future whole blood donation". In: *Transfusion* 47.8 (2007), pp. 1514–1523. DOI: https://doi.org/10.1111/j.1537-2995.2007.01292.x.

[Cus+14] B. Custer et al. "Predictors of hemoglobin recovery or deferral in blood donors with an initial successful donation". In: *Transfusion* 54.9 (2014), pp. 2267–2275.

[Fok18] J Fokkinga. "Modelling hemoglobin levels of blood donors". Master's thesis. Erasmus University Rotterdam, 2018.

[Ge+19] Tian Ge et al. "Polygenic prediction via Bayesian regression and continuous shrinkage priors". In: *Nature communications* 10.1 (2019), pp. 1–10.

[HBN98] D. Halperin, J. Baetens, and B. Newman. "The effect of short-term, temporary deferral on future blood donation". In: *Transfusion* 38.2 (1998), pp. 181–183. DOI: https://doi.org/10.1046/j.1537-2995.1998.38298193102.x.

[Hec81] James J. Heckman. "Heterogeneity and State Dependence". In: *Studies in Labor Markets*. University of Chicago Press, 1981, pp. 91–140. URL: http://www.nber.org/chapters/c8909.

[HG11] Matthew D. Hoffman and Andrew Gelman. *The No-U-Turn Sampler: Adaptively Setting Path Lengths in Hamiltonian Monte Carlo*. 2011. arXiv: 1111.4246 [stat.CO].

[Hil+11] Tessa Hillgrove et al. "The impact of temporary deferral due to low hemoglobin: future return, time to return, and frequency of subsequent donation". In: *Transfusion* 51.3 (2011), pp. 539–547. DOI: https://doi.org/10.1111/j.1537-2995.2010.02881.x.

[Inc20] Docker Inc. *Docker*. 2020. URL: https://www.docker.com.

[Kis+15] J. E. Kiss et al. "Oral iron supplementation after blood donation: a randomized clinical trial". In: *JAMA* 313.6 (2015), pp. 575–583.

[Kot+15] S. R. Kotze et al. "Predictors of hemoglobin in Danish blood donors: results from the Danish Blood Donor Study". In: *Transfusion* 55.6 (2015), pp. 1303–1311.

[Kuh20] Max Kuhn. *caret: Classification and Regression Training*. R package version 6.0-86. 2020. URL: https://CRAN.R-project.org/package=caret.

[Lob+19] Muriel Lobier et al. "The effect of donation activity dwarfs the effect of lifestyle, diet and targeted iron supplementation on blood donor iron stores". In: *PloS one* 14.8 (2019), e0220862.

[Lob+20] Muriel Lobier et al. "FinDonor 10 000 study: a cohort to identify iron depletion and factors affecting it in Finnish blood donors". In: *Vox sanguinis* 115.1 (2020), pp. 36–46.

[LW02] Andy Liaw and Matthew Wiener. "Classification and Regression by randomForest". In: *R News* 2.3 (2002), pp. 18–22.

[Nas16] K Nasserinejad. "Modeling Longitudinal Data of Blood Donors". PhD dissertation. Erasmus University Rotterdam, 2016.

[SRS21] Marloes L.C. Spekman, Steven Ramondt, and Maike G. Sweegers. "Whole blood donor behavior and availability after deferral: Consequences of a new ferritin monitoring policy". In: *Transfusion* 61.4 (2021), pp. 1112–1121. DOI: https://doi.org/10.1111/trf.16235.

[Ste09] Dan Steinberg. “Classification and Regression Trees”. In: *The Top Ten Algorithms in Data Mining.* Ed. by Xindong Wu and Vipin Kumar. Chapman & Hall/CRC, 2009. Chap. 10, pp. 179–201.

[TA19] Terry Therneau and Beth Atkinson. *rpart: Recursive Partitioning and Regression Trees.* R package version 4.1-15. 2019. URL: https://CRAN.R-project.org/package=rpart.

[Tan+05] P. Tan et al. *Introduction to Data Mining.* Addison Wesley, 2005.

[TE93] Robert J Tibshirani and Bradley Efron. “An introduction to the bootstrap”. In: *Monographs on statistics and applied probability* 57 (1993), pp. 1–436.

[Woo05] Jeffrey M. Wooldridge. “Simple solutions to the initial conditions problem in dynamic, nonlinear panel data models with unobserved heterogeneity”. In: *Journal of Applied Econometrics* 20.1 (2005), pp. 39–54. DOI: 10.1002/jae.770.
